# Minimally inconsistent reasoning in Semantic Web

**DOI:** 10.1371/journal.pone.0181056

**Published:** 2017-07-27

**Authors:** Xiaowang Zhang

**Affiliations:** 1 School of Computer Science and Technology, Tianjin University, Tianjin, China; 2 Tianjin Key Laboratory of Cognitive Computing and Application, Tianjin, China; 3 Key Laboratory of Computer Network and Information Integration (Southeast University), Ministry of Education, Nanjing, China; University of Birmingham, UNITED KINGDOM

## Abstract

Reasoning with inconsistencies is an important issue for Semantic Web as imperfect information is unavoidable in real applications. For this, different paraconsistent approaches, due to their capacity to draw as nontrivial conclusions by tolerating inconsistencies, have been proposed to reason with inconsistent description logic knowledge bases. However, existing paraconsistent approaches are often criticized for being too skeptical. To this end, this paper presents a non-monotonic paraconsistent version of description logic reasoning, called *minimally inconsistent reasoning*, where inconsistencies tolerated in the reasoning are minimized so that more reasonable conclusions can be inferred. Some desirable properties are studied, which shows that the new semantics inherits advantages of both non-monotonic reasoning and paraconsistent reasoning. A complete and sound tableau-based algorithm, called *multi-valued tableaux*, is developed to capture the minimally inconsistent reasoning. In fact, the tableaux algorithm is designed, as a framework for multi-valued DL, to allow for different underlying paraconsistent semantics, with the mere difference in the clash conditions. Finally, the complexity of minimally inconsistent description logic reasoning is shown on the same level as the (classical) description logic reasoning.

## 1 Introduction

Description logics (DLs) [1] are a family of formal knowledge representation languages, the logic formalism originally for Frame-based systems and Semantic Networks, and recently for Web Ontology Language (OWL) in Semantic Web. However, logically formalized knowledge is rarely perfect due to modeling errors, migration from other formalisms, or evolution of ontologies [2–6]. So it is unrealistic to expect that real DL ontologies are always logically consistent and complete [7]. However, DLs, as a fragment of first-order logic, allow to draw arbitrary, unsupported conclusions over inconsistent knowledge bases (KBs). Therefore, non-classical reasoning of DLs has received extensive interests [3, 5, 7–30] in recent years.

To reason with “imperfect” DL KBs, two kinds of non-classical reasoning mechanisms, namely *paraconsistent reasoning* and *non-monotonic reasoning*, have been proposed in DLs. The first applies paraconsistent semantics to DLs to tolerate inconsistent knowledge, e.g., based on Belnap’s four-valued semantics [21, 23], Kleene’s three-valued semantics [5], a variant of Belnap’s four-valued semantics [19] which is the {∨, ∧, ∼ } fragment of Nelson’s paraconsistent logic N4 [31], and Besnard & Hunter’s quasi-classical semantics [28, 30]. While these paraconsistent semantics can tolerate inconsistencies [11], they still have shortcomings. For illustration, consider a KB $K_{a}$ about “animal” and its axioms in DL syntax shown in Table 1.

**Table 1:** An example: Animal KB.

Assertions	DL-axioms
Every animal which can fly has wings	*φ*_1_: *Fly* ⊑ *HasWing*
Every animal which eats fish is a piscivore	*φ*_2_: ∃ *Eat*.*Fish* ⊑ *Piscivore*
*tweety* is not an abnormal bird or cannot fly	*φ*_3_: (¬*AbnBird* ⊔ ¬*Fly*)(*tweety*)
*ursidae* eats *salmon*	*φ*_4_: *Eat*(*ursidae*, *salmon*)
*salmon* is some fish	*φ*_5_: *Fish*(*salmon*)
*ursidae* is not a piscivore	*φ*_6_: ¬*Piscivore*(*ursidae*)

It is not hard to show that $K_{a}$ is inconsistent. This can be handled in a paraconsistent logic (e.g., four-valued logic [32]) but $K_{a}$ would have two models: Model_1_ satisfying both *AbnBird*(*tweety*) and ¬*Fly*(*tweety*) and Model_2_ satisfying both ¬*AbnBird*(*tweety*) and *Fly*(*tweety*). However, in commonsense reasoning (such as causal reasoning), it is widely accepted that Model_1_ is not intended since the inconsistency of $K_{a}$ is mainly caused by the assertion ¬*Piscivore*(*ursidae*) and a consequence *Piscivore*(*ursidae*), which is inferred from three assertions (*φ*_2_, *φ*_4_, *φ*_5_), and the part of $K_{a}$ in representing whether *tweety* can fly is consistent.

To enable the desired reasoning mechanism, non-monotonic reasoning is required because some inferences should be blocked when information increased. Non-monotonic reasoning takes inconsistency as “exception” in reasoning [33]. Different treatments with “exception” lead to different non-monotonic systems, e.g., Reiter’s default logic [7], epistemic operators [12, 13], McCarthy’s circumscription [10], and Motik and Rosati’s MKNF (Minimal Knowledge and Negation as Failure) [13]. Accordingly, existing non-monotonic DLs are achieved by extending classical DL syntax with extra operators or restricting some DL constructors, e.g., via modal operators [12, 13], via open default rules in [7], or using circumscription patterns [10, 34]. However, existing non-monotonic DL systems still have limitations in inconsistency handling because their underlying logics have to be monotonic. In other words, they can no longer work if the new knowledge contains inconsistency, as the second situation in the illustrative example above. Indeed, the underlying monotonic logic for non-monotonic logics is not necessarily classical, but should be chosen according to the intended applications instead [35]. If, for example, we do not totally reject inconsistent knowledge, then an underlying paraconsistent logic might be a better choice than the classical logic.

As argued above, paraconsistent reasoning and non-monotonic reasoning have their advantages and disadvantages. Combining paraconsistent and non-monotonic reasoning has been studied for several systems [36–38], but the challenge of a paraconsistent non-monotonic DL system exists in the preservation of desired features of classical DLs. Ideally, such a combined reasoning should satisfy the following properties: (1) It works on original DL syntax; (2) It is paraconsistent; (3) It is non-monotonic; (4) It is decidable and does not bring extra computational complexity compared to classical DLs.

To resolve this issue for paraconsistent reasoning and non-monotonic reasoning in DL ontologies, in this paper, we propose to use minimal models in paraconsistent description logics. As a result, we introduce a minimally inconsistent description logic satisfying all of these requirements, which can minimize the inconsistent part in KB in order to infer more feasible information. This work is a significant extension of the preliminary results that were prevously published in [39, 40] where [39] presents a paradoxical semantics (as a class of complete multi-valued semantics) for DL and [40] presents a minimally paradoxical semantics (as a class of minimally complete multi-valued semantics) for DLs, respectively. In this paper, we generate paradoxical semantics and minimally paradoxical semantics for DLs in a unified framework so that we could investigate general properties and relations with other classes of MVDL and MIDL, such as four-valued semantics and minimally four-valued semantics. Moreover, we develop a tableaux called multi-valued tableaux to characterize the minimal inconsistent reasoning with DLs. Our main contributions are summarized as follows:

- We define two sorts of the minimally inconsistent DL reasoning based on two multi-valued semantics for DLs;
- We propose a new framework of tableaux called multi-valued tableaux as the proof systems for both multi-valued DLs and minimally inconsistent DL reasonings.
- By the proposed tableaux algorithm for minimally inconsistent DL reasonings, we show that both of the our minimally inconsistent DL reasoning problems are no harder than their corresponding classical DL reasoning problems.

The rest of the paper is organized as follows: a brief review of DL is first introduced, and then Section 3 defines multi-valued DL. Section 4 proposes the minimally inconsistent DL reasoning and Section 5 presents multi-valued tableaux framework. Section 6 discusses related works and Section 7 concludes the paper. All proofs are presented in Appendix.

## 2 Preliminaries

In this section, we give a brief introduction of description logics and paraconsistent logic.

### 2.1 Description logics

In DLs, elementary descriptions are *concept names* and *role names*. Complex descriptions are built from them inductively using concept and role constructors provided by the particular DL in consideration. For a comprehensive understanding, we refer readers to the Description Logic Handbook [1].

In this paper, we consider the ALC which is a simple yet relatively expressive DL. Let *N*_*C*_ and *N*_*R*_ be pairwise disjoint and countably infinite sets of *concept names* and *role names* respectively. Let *N*_*I*_ be an infinite set of *individual names*. We use the letters *A*, *B* for concept names, the letter *R*, *S* for role names, the letters *C*, *D* for concepts, and the letters *a*, *b* for individual names. Let ⊤ and ⊥ denote the *top concept* and the *bottom concept* respectively. The set of ALC concepts is the smallest set such that:

- every concept name is a concept;
- if *C*, *D* are concepts, *R* is a role name, then the following expressions are also concepts: ¬*C* (*full negation*), *C*⊓*D* (*concept conjunction*), *C*⊔*D* (*concept disjunction*), ∀*R*.*C* (*value restriction on role name*) and ∃*R*.*C* (*existential restriction on role name*).

For instance, the concept description *Student* ⊔ *Staff* is an ALC-concept describing those members that are students or staffs. Suppose *HasCourse* is a role name, the concept description *Student* ⊓ ∃*hasCourse.ComputerScience* expresses those students who have some courses of computer science. The concept description ∀*hasCourse*.⊥ ⊓ *Staff* describes those staffs who have no course.

An interpretation $I=(\Delta^{I},\cdot^{I})$ consists of a non-empty domain $\Delta^{I}$ and a mapping $\cdot^{I}$ which maps every concept to a subset of $\Delta^{I}$ and every role to a subset of $\Delta^{I}\times \Delta^{I}$ such that the following conditions are satisfied:

- ${⊤}^{I}=\Delta^{I}$;
- ${⊥}^{I}=\emptyset$;
- ${\left(\neg C\right)}^{I}=\Delta^{I}\setminus C^{I}$;
- ${\left(C⊓D\right)}^{I}=C^{I}\cap D^{I}$;
- ${\left(C⊔D\right)}^{I}=C^{I}\cup D^{I}$;
- ${\left(\exists R.C\right)}^{I}=\left\{x|\exists y.\;\left(x,y\right)\in R^{I}\;\&\;y\in C^{I}\right\}$; and
- ${\left(\forall R.C\right)}^{I}=\left\{x|\forall y.\;\left(x,y\right)\in R^{I}\Rightarrow y\in C^{I}\right\}$.

Here & means “and” and ⇒ means “imply”.

A *general concept inclusion axiom* (GCI) or *a terminological axiom* is an inclusion statement of the form *C* ⊑ *D*, where *C* and *D* are two (possibly complex) ALC concepts (concepts for short). It is the statement about how concepts are related to each other. An interpretation I satisfies a GCI *C* ⊑ *D* if $C^{I}\subseteq D^{I}$, denoted by I⊨C⊑D. A finite set of GCIs is called a *TBox*. We can also formulate statements about individuals. A *concept* (*role*) *assertion* has the form *C*(*a*) (*R*(*a*, *b*)). An *ABox* consists of a finite set of concept assertions and role assertions. Concept assertions, role assertions and GCIs are *axioms*. In an ABox, each axiom describes a specific fact of an application domain in terms of concepts and roles. To give a semantics to ABoxes, we need to extend interpretations to individual names. For each individual name *a*, $\cdot^{I}$ maps it to an element $a^{I}\in \Delta^{I}$. An interpretation I satisfies *C*(*a*) if $a^{I}\in C^{I}$, denoted by I⊨C(a). I satisfies *R*(*a*, *b*) if $\left(a^{I},b^{I}\right)\in R^{I}$, denoted by I⊨R(a,b). A knowledge base (KB) K consists of a TBox and an ABox. An interpretation I is a *model* of a DL (TBox or ABox) axiom if it satisfies this axiom, and it is a model of a KB K if it satisfies every axiom in K. Mod(K) denotes the collection of all models of K.

A concept *D* *subsumes* a concept *C* with respect to (w.r.t.) a TBox T if each model of T is a model of axiom *C* ⊑ *D*. Moreover, two concepts *C*, *D* are *equivalent*, denoted by C≜D, if for any interpretation I such that $C^{I}=D^{I}$. Indeed, C≜D if and only if I⊨C⊑D and I⊨D⊑C for any interpretation I. A KB K is *consistent* if there exists some model of K, i.e., Mod(K)≠∅; *inconsistent* otherwise.

Given a KB K and an axiom *φ*, we say K *entails* *φ*, denoted as K⊨ϕ, if every model of K is a model of *φ*, i.e., Mod(K)⊆Mod({φ}). Note that DL reasoning is realized via the entailment relation ⊨. A concept *C* is *satisfiable* w.r.t. a TBox T if there exists a model I of T such that $C^{I}\neq \emptyset$; and *unsatisfiable* otherwise.

Two basic reasoning problems, namely, *instance checking* (checking whether an individual is an instance of a given concept) and *subsumption checking* (checking whether a concept subsumes a given concept) can be reduced to the problem of consistency. That is,

**Lemma 1** ([1]) Let K be a KB, *C*, *D* concepts and *a* an individual in ALC. Then

1. K⊨C(a) if and only if K∪{¬C(a)} is inconsistent;
2. K⊨C⊑D if and only if K∪{C⊓¬D(ι)} is inconsistent where *ι* is a new individual not occurring in K.

In ALC, the problem of checking consistency of an ABox is PSPACE-Complete and the problem of checking consistency of an ABox w.r.t. a general TBox is EXPTIME-Complete [1].

For some inference rules in propositional logic, their counterparts in DLs still hold as follows: let *C*, *D*, *E* be DL concepts and $K,K_{1},K_{2},K$ be KBs,

- *modus ponens* (**MP**): {*C*(*a*), *C* ⊑ *D*}⊨*D*(*a*);
- *modus tollens* (**MT**): {¬*D*(*a*), *C* ⊑ *D*}⊨*C*(*a*);
- *disjunctive syllogism* (**DS**): {¬*C*(*a*), *C* ⊔ *D*(*a*)} ⊨ *D*(*a*);
- *resolution* (**R**): {*C* ⊔ *D*(*a*), ¬*C*⊔*E*(*a*)} ⊨ *D* ⊔ *E*(*a*);
- *disjunction introduction* (**DI**): {*C*(*a*)} ⊨ *C* ⊔ *D*(*a*);
- *implication* (**I**): K⊨C⊑D if and only if K⊨¬C⊔D(a) for any *a*;
- *transitive inclusion* (**TI**) if K⊨C⊑D and K⊨D⊑E then K⊨C⊑E;
- *excluded middle* (**EM**): ∅ ⊨ *C* ⊔ ¬*C*(*a*);
- *transitivity* (**T**): if $K_{1}⊨K_{2}$ and $K_{2}⊨K_{3}$ then $K_{1}⊨K_{3}$;
- *monotonicity* (**M**): if $K_{1}⊨K_{2}$ then $K_{3}⊨K_{2}$ where $K_{1}\subseteq K_{3}$.

The property of **DS** is a special case of the resolution rule. The property of **I** can be used to reduce the subsumption problem to the satisfaction problem.

We say an entailment relation ⊨_*x*_ satisfies a property **P** above if it is true when ⊨ is replaced by ⊨_*x*_. If the entailment of a logic satisfies the property of monotonicity, we say *monotonic logic*; and otherwise *non-monotonic logic*. Clearly, all members of the DL family satisfy properties listed above and in particular, each DL is a monotonic logic.

Given an axiom *φ*, if for any interpretation I, I⊨φ then *φ* is a *tautology*; and if for any interpretation I, I⊭φ, *φ* is a *contradiction*. For instance, the three axioms ⊥ ⊑ ⊤, ⊤(*a*), and *A* ⊔ ¬*A*(*a*) are tautologies while the three axioms ⊤ ⊑ ⊥, ⊥(*a*), and *A* ⊓ ¬*A*(*a*) are contradictions. Indeed, the property of **EM** captures a sort of tautologies among many others.

### 2.2 Paraconsistent logic

In the last of this section, we briefly recall paraconsistent logic and paraconsistent description logic. More details can be found in [30].

In practical reasoning, it is common that there exists “too much” information (classically inconsistent information) about some situation. However, the reasoning of classical logic would be trivialized when inconsistent information exists due to a curious feature, known as *the principle of explosion* or (*ex falso quodlibet*) can be expressed formally as: for any formula *φ*, *ψ*, {*φ*, ¬*φ*} ⊨ *ψ*.

This is the need to derive reasonable inferences without deriving the trivial inferences that follow the ex falso quodlibet. In other words, we need a logic, called *paraconsistent logic* (or *inconsistency-tolerant logic*) where the principle of explosion fails in its reasoning [41].

Description logic fails to be paraconsistent because an inconsistent KB K does not possess any model, i.e., Mod(K)=∅. In this sense, we say that the entailment ⊨ satisfies the principle of explosion. Thus, the entailment of a paraconsistent description logic does not satisfy the principle of explosion, called a *paraconsistent entailment*.

Indeed, if some properties about inference are allowed together, the inference with inconsistent knowledge possibly becomes explosive.

For instance, let ⊨_*p*_ be an entailment. Assume that ⊨_*p*_ satisfies DS, DI and transitivity. Given an ABox $A_{1}=\left\{A\left(a\right),\neg A\left(a\right)\right\}$, $A_{1}$ is inconsistent. Because ⊨_*p*_ satisfies DI, $A_{1}{⊨}_{p}A⊔B\left(a\right)$ for arbitrary *B*. Let $A_{2}=\left\{A\left(a\right),\neg A\left(a\right),\left(A⊔B\right)\left(a\right)\right\}$. We conclude that $A_{1}{⊨}_{p}A_{2}$. Because ⊨_*p*_ satisfies DS, we conclude that $A_{2}{⊨}_{p}B\left(a\right)$. Then, {*A*(*a*), ¬*A*(*a*)} ⊨ _*p*_ *B*(*a*) for any *B* since ⊨_*p*_ satisfies transitivity. In this sense, ⊨_*p*_ loses a property so-called “*relevance*”, which requires sharing of variables between premises and conclusion, in relevance logic [42].

A feasible method to make the principle of explosion invalid is weakening inference power by prohibiting some inference rules in reasoning [3].

The following lemma presents a common feature of all paraconsistent description logics.

**Lemma 2** A paraconsistent entailment does not satisfy DS, DI and transitivity.

## 3 Multi-valued description logic

To construct a paraconsistent non-monotonic ALC, we first revise paraconsistent ALC in this section in two steps: (1) introducing a general framework, called multi-valued description logic (MVDL); and (2) constructing two restricted versions, namely, four-valued DL [21, 43] and paradoxical DL, in MVDL [39].

### 3.1 Syntax and semantics of MVDL

Syntactically, MVDL revises ALC in two ways:

- To block the conflict between positive and negative information, four-valued DL and paradoxical DL do not treat the negation of a concept as its “opposite” concept, but allow them to have a common individual. As shown in [19, 21, 28], to maintain the transformation from entailment to inconsistency checking (cf. Proposition 4), a stronger negation should be introduced, called *complement* of a concept *C*, denoted $\bar{C}$. So MVDL contains $\bar{\;\cdot \;}$ as an extra concept constructor.
- As stated in [21], we allow for three kinds of concept inclusions, namely, *material inclusion* (*C* ↦ *D*), *internal inclusion* (*C* ⊏ *D*) and *strong inclusion* (*C* → *D*).

The semantics of MVDL is based on four-valued semantics, called *base interpretations*.

**Definition 1** [21] A *base interpretation* I is a pair $\left(\Delta^{I},\cdot^{I}\right)$ where the domain $\Delta^{I}$ is a set of individuals, and the assignment function $\cdot^{I}$ assigns each individual to an element of $\Delta^{I}$, assigns each concept name *A* to $\langle {\text{p}}^{+}\left(A^{I}\right),{\text{p}}^{-}\left(A^{I}\right)\rangle$ where ${\text{p}}^{+}\left(A^{I}\right),{\text{p}}^{-}\left(A^{I}\right)\subseteq \Delta^{I}$, each role name *R* to $\langle {\text{p}}^{+}\left(R^{I}\right),{\text{p}}^{-}\left(R^{I}\right)\rangle$ where ${\text{p}}^{+}\left(R^{I}\right),{\text{p}}^{-}\left(R^{I}\right)\subseteq \Delta^{I}\times \Delta^{I}$ and satisfies the followings:

$$\begin{array}{c} \begin{array}{ccc} {⊥}^{I} & = & \langle \emptyset^{I},\Delta^{I}\rangle ;\\ {⊤}^{I} & = & \langle \Delta^{I},\emptyset^{I}\rangle ;\\ {\left(\neg C\right)}^{I} & = & \langle {\text{p}}^{-}\left(C^{I}\right),{\text{p}}^{+}\left(C^{I}\right)\rangle ;\\ {\left(\bar{C}\right)}^{I} & = & \langle \Delta^{I}\setminus {\text{p}}^{+}\left(C^{I}\right),\Delta^{I}\setminus {\text{p}}^{-}\left(C^{I}\right)\rangle ;\\ {\left(C⊓D\right)}^{I} & = & \langle {\text{p}}^{+}\left(C^{I}\right)\cap {\text{p}}^{+}\left(D^{I}\right),{\text{p}}^{-}\left(C^{I}\right)\cup {\text{p}}^{-}\left(D^{I}\right)\rangle ;\\ {\left(C⊔D\right)}^{I} & = & \langle {\text{p}}^{+}\left(C^{I}\right)\cup {\text{p}}^{+}\left(D^{I}\right),{\text{p}}^{-}\left(C^{I}\right)\cap {\text{p}}^{-}\left(D^{I}\right)\rangle ;\\ {\left(\exists R.C\right)}^{I} & = & \langle \{x|\exists y.\;(x,y)\in S\;\wedge \;y\in P\},\{x|\forall y.\;(x,y)\in S\Rightarrow y\in N\}\rangle ;\\ {\left(\forall R.C\right)}^{I} & = & \langle \{x|\forall y.\;(x,y)\in S\Rightarrow y\in P\},\{x|\exists y.\;(x,y)\in S\wedge y\in N)\}\rangle . \end{array} \end{array}$$

where $C^{I}=\langle {\text{p}}^{+}\left(C^{I}\right),{\text{p}}^{-}\left(C^{I}\right)\rangle$, $D^{I}=\langle {\text{p}}^{+}\left(D^{I}\right),{\text{p}}^{-}\left(D^{I}\right)\rangle$, and $R^{I}=\langle {\text{p}}^{+}\left(R^{I}\right)$, ${\text{p}}^{-}\left(R^{I}\right)\rangle$.

**Remark** **1**

1. Two arguments of a base interpretation of a concept/role are not necessarily disjoint with respect to the domain.
2. In MVDL ALC, the second argument of every interpretation of a role is not necessary. However, we still reserve it here in order that the semantics of MVDL can directly extend more expressive DLs such as OWL 2 where the negation of a role ¬*R* is also considered as a role [44], which takes the same treatment as in four-valued DLs [43] or quasi-classical DLs [30].
3. The first argument of a base interpretation of a concept is a set of elements known to be in the extension of concept and the second argument is a set of elements known to be in the extension of the negation of the concept. In this sense, an element is unknown to be in the extension of a concept (i.e., no information is given), which is different from that an element is known to be in the extension of the negation of a concept in an open world.

For instance, let I be a base interpretation with domain {*tweety*, *ursidae*}, and assigning *Fly* a pair $\langle \left\{{\text{tweety}}^{I}\right\},\left\{{\text{ursidae}}^{I}\right\}\rangle$. Then I tells us that it is known that *tweety* can fly and *ursidae* can not fly.

Two extended concepts *C*, *D* are *multi-valued equivalent*, denote by $C{≜}_{m}D$, if for each base interpretation I, we have $C^{I}=D^{I}$, that is, ${\text{p}}^{+}\left(C^{I}\right)={\text{p}}^{+}\left(D^{I}\right)$ and ${\text{p}}^{-}\left(C^{I}\right)={\text{p}}^{-}\left(D^{I}\right)$. Then, the negation ¬ and the complement $\bar{\;\cdot \;}$ satisfy following properties.

**Proposition 1** [43] Let *C*, *D* be concepts and *R* a role in MVDL.

1. $\neg \bar{C}{≜}_{m}\bar{\neg C}$;
2. $\neg \left(\neg C\right){≜}_{m}C$ and $\bar{\bar{C}}{≜}_{m}C$;
3. $\neg \left(C⊓D\right){≜}_{m}\neg C⊔\neg D$ and $\bar{C⊓D}{≜}_{m}\bar{C}⊔\bar{D}$;
4. $\neg \left(C⊔D\right){≜}_{m}\neg C⊓\neg D$ and $\bar{C⊔D}{≜}_{m}\bar{C}⊓\bar{D}$;
5. $\neg \exists R.C{≜}_{m}\forall R.\neg C$ and $\bar{\exists R.C}{≜}_{m}\forall R.\bar{C}$;
6. $\neg \forall R.C{≜}_{m}\exists R.\neg C$ and $\bar{\forall R.C}{≜}_{m}\exists R.\bar{C}$.

Note that (*C* ⊔ ¬*C*) 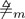 ⊤ and (*C* ⊓ ¬*C*) 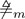 ⊥ for any *C*. For instance, let Δ = {*a*, *b*, …,} and I a base interpretation on Δ such that $C^{I}=\langle \left\{a^{I}\right\},\left\{a^{I}\right\}\rangle$. Thus ${\left(C⊔\neg C\right)}^{I}=\langle \left\{a^{I}\right\},\left\{a^{I}\right\}\rangle$ while ${⊤}^{I}=\langle \Delta^{I},\emptyset \rangle$. Moreover, ${\left(C⊓\neg C\right)}^{I}=\langle \left\{a^{I}\right\},\left\{a^{I}\right\}\rangle$ while ${⊥}^{I}=\langle \emptyset ,\Delta^{I}\rangle$. Then (*C* ⊔ ¬*C*) 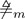 ⊤ and (*C* ⊓ ¬*C*) 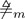 ⊥.

**Definition 2** [21] Let *C*, *D* be two extended concepts, *R* a role, and I a base interpretation. The multi-valued satisfaction between a base interpretation I and an extended axiom *φ*, denoted by $I{⊨}_{m}\varphi$, is defined as follows:

1. $I{⊨}_{m}C\left(a\right)$, if $a^{I}\in {\text{p}}^{+}\left(C^{I}\right)$;
2. $I{⊨}_{m}R\left(a,b\right)$, if $\left(a^{I},b^{I}\right)\in {\text{p}}^{+}\left(R^{I}\right)$;
3. $I{⊨}_{m}C\mapsto D$, if $\Delta^{I}\setminus {\text{p}}^{-}\left(C^{I}\right)\subseteq {\text{p}}^{+}\left(D^{I}\right)$;
4. $I{⊨}_{m}C⊏D$, if ${\text{p}}^{+}\left(C^{I}\right)\subseteq {\text{p}}^{+}\left(D^{I}\right)$;
5. $I{⊨}_{m}C\to D$, if ${\text{p}}^{+}\left(C^{I}\right)\subseteq {\text{p}}^{+}\left(D^{I}\right)$ and ${\text{p}}^{-}\left(D^{I}\right)\subseteq {\text{p}}^{-}\left(C^{I}\right)$.

In Definition 2, the multi-valued satisfaction of *C*(*a*) (or *R*(*a*, *b*)) means that *a* (or (*a*, *b*)) is known not in *C* (or *R*); the satisfaction of *C* ↦ *D* means that each instance known not in ¬*C* must be in *D*; the satisfaction of *C* ⊏ *D* means that each instance known to be in *C* must be in *D*; and the satisfaction of *C* → *D* means that each instance known to be in *C* (resp. ¬*D*) must be in *D* (resp. ¬*C*).

By Definition 2, it follows that the multi-valued satisfaction of *C* → *D* can be represented by the multi-valued satisfaction of *C* ⊏ *D* and ¬*D* ⊏ ¬*C*. It is easy to observe that for any two concepts *C*, *D* for any base interpretation I, we have $I{⊨}_{m}C\to D$ if and only if $I{⊨}_{m}C⊏D$ and $I{⊨}_{m}\neg D⊏\neg C$.

Let K be an extended KB and I a base interpretation. I is a *multi-valued model* of K if $I{⊨}_{m}\varphi$ for any φ∈K. Let $Mod_{m}\left(K\right)$ denote the collection of all multi-valued models of K. K is *multi-valued consistent* if $Mod_{m}\left(K\right)\neq \emptyset$; and *multi-valued inconsistent* otherwise. The *multi-valued entailment* ⊨_*m*_ is defined as: $K_{1}{⊨}_{m}K_{2}$ if $Mod_{m}\left(K_{1}\right)\subseteq Mod_{m}\left(K_{2}\right)$.

In MVDL, a classical inconsistent KB has some multi-valued models since the negation ¬ is weaker under the multi-valued semantics. For instance, let $A^{\prime }=\left\{A\left(a\right),\neg A\left(a\right)\right\}$ be an ABox. Assume that Δ = {*a*, …} and I is a base interpretation on Δ such that $A^{I}=\langle \left\{a^{I}\right\},\left\{a^{I}\right\}\rangle$. Thus $I{⊨}_{m}A\left(a\right)$ and $I{⊨}_{m}\neg A\left(a\right)$. However, not all extended KBs have some multi-valued model. For example, when $A=\{A\left(a\right),\bar{A}\left(a\right)\}$, there exists no multi-valued model of A since ${\text{p}}^{+}\left(A^{I}\right)\cap {\text{p}}^{+}\left({\bar{A}}^{I}\right)=\emptyset$ for any base interpretation I.

**Example 1** Let’s consider the KB $K_{a}$ in Section 1 by selecting the internal inclusion. $K_{a}=\left(T_{a},A_{a}\right)$ where the TBox $T_{a}=\left\{\text{Fly}⊏\text{HasWing},\exists \;\text{Eat}.\text{Fish}⊏\text{Piscivore}\right\}$ and the ABox $A_{a}=\{\neg \text{AbnBird}⊔\neg \text{Fly}\left(\text{tweety}\right)$, *Eat*(*ursidae*, *salmon*), *Fish*(*salmon*), ¬*Piscivore*(*ursidae*)} and Δ = {*tweety*, *ursidae*, *a*_1_, *a*_2_, *a*_3_}.

1. Let $I_{1}$ be a base interpretation where ${\text{AbnBird}}^{I_{1}}=\langle \{{\text{tweety}}^{I},a_{1}^{I_{1}}$, $a_{2}^{I_{1}}\}$, $\{a_{3}^{I_{1}}\}\rangle$, ${\text{Fly}}^{I_{1}}=\langle \{{\text{tweety}}^{I_{1}}$, $a_{3}^{I_{1}}\},\left\{{\text{tweety}}^{I_{1}},a_{1}^{I_{1}}\right\}\rangle$, ${\text{HasWing}}^{I_{1}}=\langle \{{\text{tweety}}^{I_{1}}$, $a_{3}^{I_{1}}\}$, $\{a_{1}^{I_{1}},a_{2}^{I_{1}}\}\rangle$, ${\text{Fish}}^{I_{1}}=\langle {\text{salmon}}^{I_{1}},\emptyset \rangle$, ${\text{Eat}}^{I_{1}}=\langle \left({\text{ursidae}}^{I_{1}},{\text{salmon}}^{I_{1}}\right),\emptyset \rangle$, and ${\text{Piscivore}}^{I_{1}}=\langle \left\{{\text{ursidae}}^{I_{1}}\right\},\left\{{\text{ursidae}}^{I_{1}}\right\}\rangle$.
2. Let $I_{2}$ be a base interpretation where ${\text{AbnBird}}^{I_{2}}=\langle \left\{a_{1}^{I_{2}},a_{2}^{I_{2}}\right\},\left\{{\text{tweety}}^{I_{1}},a_{3}^{I_{2}}\right\}\rangle$, ${\text{Fly}}^{I_{2}}=\langle \left\{{\text{tweety}}^{I_{1}},a_{3}^{I_{2}}\right\},\left\{a_{1}^{I_{1}},a_{2}^{I_{2}}\right\}\rangle$, ${\text{HasWing}}^{I_{2}}={\text{HasWing}}^{I_{1}}$, ${\text{Fish}}^{I_{2}}={\text{Fish}}^{I_{1}}$, ${\text{Eat}}^{I_{2}}={\text{Eat}}^{I_{1}}$, and ${\text{Piscivore}}^{I_{2}}={\text{Piscivore}}^{I_{1}}$.

Thus $I_{1}$ and $I_{2}$ are multi-valued models of $K_{a}$. Clearly, multi-valued models can certainly tolerate classical inconsistency. For instance, in the multi-valued model $I_{1}$, *tweety* is interpreted to be in both *Fly* and ¬*Fly*. Intuitively, under the multi-valued semantics of $I_{1}$, it is acceptable no matter that *tweety* can fly or not.

The nonexistence of multi-valued models leads to the principle of explosion because all conclusions are satisfied by the empty model set. For instance, {⊥(*a*)} ⊨ *φ* for any axiom *φ*. A form of KBs called *satisfiable form* is introduced by [21] by substituting ⊤ with *NA*⊔¬*NA* and ⊥ with *NA* ⊓ ¬*NA* where *NA* is a new concept name. Let SF(K) denote the satisfiable form of it.

By applying that transformation, we also conclude:

**Proposition 2** [43] For any classical KB K, $Mod_{m}\left(SF\left(K\right)\right)\neq \emptyset$.

Due to Proposition 2, we consider all classical KBs (including classically inconsistent KBs) are in satisfiable form in this paper.

Therefore, we obtain that the multi-valued entailment on KBs in satisfiable form is paraconsistent.

**Proposition 3** For any classical KB K, there exists some axiom *φ* such that $SF\left(K\right){⊭}_{m}\varphi$.

Lemma 1 for DLs can be extended in MVDL.

**Proposition 4** [43] Let K be an extended KB and *C*, *D* two extended concepts. The followings hold.

1. $K{⊨}_{m}C\left(a\right)$ if and only if $K\cup \{\bar{C}\left(a\right)\}$ is multi-valued inconsistent;
2. $K{⊨}_{m}C\mapsto D$ if and only if $K\cup \{\left(\neg \bar{C}⊓\bar{D}\right)\left(\tau \right)\}$ is multi-valued inconsistent;
3. $K{⊨}_{m}C⊏D$ if and only if $K\cup \{\left(C⊓\bar{D}\right)\left(\tau \right)\}$ is multi-valued inconsistent;
4. $K{⊨}_{m}C\to D$ if and only if both $K\cup \{\left(C⊓\bar{D}\right)\left(\tau \right)\}$ and $K\cup \{\left(\neg D⊓\neg \bar{C}\right)\left(\tau \right)\}$ are multi-valued inconsistent.

By Proposition 4, the multi-valued entailment problem can be reduced to the multi-valued consistency problem of KBs.

The following result states that MVDL inherits monotonicity of DL.

**Proposition 5** MVDL is monotonic.

Different from material inclusion, the multi-valued entailment for both internal inclusion and strong inclusion hold the property of transitive inclusion.

**Proposition 6** Let T be an extended TBox and *C*, *D*, *E* three extended concepts.

1. If $T{⊨}_{m}C⊏D$ and $T{⊨}_{m}D⊏E$ then $T{⊨}_{m}C⊏E$;
2. If $T{⊨}_{m}C\to D$ and $T{⊨}_{m}D\to E$ then $T{⊨}_{m}C\to E$.

### 3.2 Constructing four-valued DL and paradoxical DL in MVDL

We show that both four-valued DL [21] and paradoxical DL can be constructed in MVDL.

#### Constructing four-valued DL in MVDL

By the syntax and semantics of MVDL, it is not hard to show that MVDL extends four-valued DL in the following way:

1. The syntax of MVDL extends four-valued DL by introducing the complement of a concept $\bar{C}$. That is, the language of four-valued DL is a sublanguage of MVDL.
2. With the restriction of MVDL semantics to four-valued DL constructors, four-valued interpretations are identical to MVDL base interpretations. Thus, the entailment problems in four-valued DL can be characterized by their corresponding problems in MVDL.

**Proposition 7** Let K be a KB, *φ* an axiom, and, ⊨_4_ the four-valued entailment as defined in [21].

$$K{⊨}_{4}\varphi \text{ if and only if }K{⊨}_{m}\varphi .$$

Note that, by Proposition 4, the four-valued entailment problems in four-valued DL can be reduced to the multi-valued consistency problem in MVDL, with the appearance of complement constructor.

#### Constructing paradoxical DL in MVDL

The syntax of paradoxical DL [39] is the same as that of four-valued DL. And a paradoxical interpretation $I_{p}$ for paradoxical DL is a restricted four-valued interpretation in the following way:

1. for any concept *C*, ${\text{p}}^{+}\left(C^{I}\right)\cup {\text{p}}^{-}\left(C^{I}\right)=\Delta^{I}$;
2. for any role *R*, ${\text{p}}^{+}\left(R^{I}\right)\cup {\text{p}}^{-}\left(R^{I}\right)=\Delta^{I}\times \Delta^{I}$.

The paradoxical satisfaction and the paradoxical entailment ⊨_*LP*_ are analogously defined in paradoxical DL by using paradoxical interpretations.

To construct paradoxical DL in MVDL, we introduce the notion of *complete base interpretation*. A base interpretation I is *complete* if I satisfies two above-mentioned conditions. Intuitively, two arguments of complete base interpretations are not necessarily disjoint but the union of two arguments fills the whole domain.

Different from general base interpretations, complete base interpretations can represent tautologies.

**Proposition 8** Let *C* be a concept and I be a base interpretation. If I is complete then

$$\begin{array}{c} {\text{p}}^{+}{\left(C⊔\neg C\right)}^{I}={\text{p}}^{+}\left({⊤}^{I}\right). \end{array}$$ (1)

Let K be an extended KB and I a base interpretation, I is a *complete multi-valued model* of K if I is complete and I is a multi-valued model of K, written $I{⊨}_{m_{c}}K$. And let $Mod_{m_{c}}\left(K\right)$ denote the collection of all complete multi-valued models of K. We say that K is *complete multi-valued consistent* if $Mod_{m_{c}}\left(K\right)\neq \emptyset$; and *complete multi-valued inconsistent* otherwise.

Consider an extended ABox $A=\{\bar{A}\left(a\right),\neg \bar{A}\left(a\right)\}$. If $I\in Mod_{m_{c}}\left(K\right)$, it holds that $a^{I}\in \Delta^{I}\setminus {\text{p}}^{+}\left(A^{I}\right)$ and $a^{I}\in \Delta^{I}\setminus {\text{p}}^{-}\left(A^{I}\right)$. Thus $a^{I}\in \Delta^{I}\setminus ({\text{p}}^{+}\left(A^{I}\right)\cup {\text{p}}^{-}\left(A^{I}\right)$. Then $({\text{p}}^{+}\left(A^{I}\right)\cup {\text{p}}^{-}\left(A^{I}\right)\neq \Delta^{I}$, contradicting with the fact that I is complete. Therefore, A is complete multi-valued inconsistent. However, all classical ALC KBs in satisfiable form are complete multi-valued consistent.

**Proposition 9** For any KB K, $Mod_{m_{c}}\left(SF\left(K\right)\right)\neq \emptyset$.

For instance, ABox $A^{\prime }=\left\{A\left(a\right),\neg A\left(a\right)\right\}$ has a complete multi-valued model I with Δ = {*a*, …} and $A^{I}=\langle \left\{a^{I}\right\},\Delta^{I}\rangle$.

The *complete multi-valued entailment*, denoted by ⊨_*m*_*c*__, is defined as follows: let $K_{1},K_{2}$ be two extended KBs, $K_{1}{⊨}_{m_{c}}K_{2}$ if $Mod_{m_{c}}\left(K_{1}\right)\subseteq Mod_{m_{c}}\left(K_{2}\right)$. Analogously, we can show that the complete multi-valued entailment ⊨_*m*_*c*__ is paraconsistent in KBs by Proposition 9.

Based on the discussion above, we directly conclude the following proposition:

**Proposition 10** Let K be a KB and *φ* an axiom. $K{⊨}_{LP}\varphi$ if and only if $K{⊨}_{m_{c}}\varphi$.

Analogously, the complete multi-valued entailment can be reduced to the complete multi-valued consistency problem in KBs.

**Proposition 11** Let K be a KB and *C*, *D* two concepts. The followings hold.

1. $K{⊨}_{m_{c}}C\left(a\right)$ if and only if $K\cup \{\bar{C}\left(a\right)\}$ is multi-valued inconsistent;
2. $K{⊨}_{m_{c}}C\mapsto D$ if and only if $K\cup \{\left(\neg \bar{C}⊓\bar{D}\right)\left(\tau \right)\}$ is complete multi-valued inconsistent;
3. $K{⊨}_{m_{c}}C⊏D$ if and only if $K\cup \{\left(C⊓\bar{D}\right)\left(\tau \right)\}$ is complete multi-valued inconsistent;
4. $K{⊨}_{m_{c}}C\to D$ if and only if both $K\cup \{\left(C⊓\bar{D}\right)\left(\tau \right)\}$ and $K\cup \{\left(\neg D⊓\neg \bar{C}\right)\left(\tau \right)\}$ are complete multi-valued inconsistent.

In general, the material inclusion does not always satisfy the property of transitive inclusion under ⊨_*m*_*c*__ as under ⊨_*m*_. However, it holds the transitive inclusion property when restricted to *conflict-free* concepts. An extended concept is *C* is called *conflict-free* with respect to an extended KB *K* if ${\text{p}}^{+}\left(C^{I}\right)=\Delta^{I}\setminus {\text{p}}^{-}\left(C^{I}\right)$ for any complete multi-valued model I of K.

**Proposition 12** Let T be an extended TBox and *C*, *D*, *E* three extended concepts. If *D* is conflict-free with respect to T, then $T{⊨}_{m_{c}}C\mapsto D$ and $T{⊨}_{m_{c}}D\mapsto E$ imply $T{⊨}_{m_{c}}C\mapsto E$.

Proposition 12 shows that information can be properly propagated even if there are conflicts. A further advantage of the complete multi-valued entailment is that it holds the principle of excluded middle.

**Proposition 13** For any concept *C* and any individual *a*, ∅ ⊨_*m*_*c*__ *C* ⊔ ¬*C*(*a*).

However, Proposition 13 does not hold in extended concepts. For instance, let Δ be a domain and I a base interpretation such that $A^{I}=\langle \left\{a\right\},\Delta^{I}\rangle$. We can conclude that I is a complete multi-valued model of ∅ while I is not a complete multi-valued model of $\left(A⊔\bar{A}\right)\left(a\right)$ since ${\text{p}}^{+}\left({\bar{A}}^{I}\right)\cup {\text{p}}^{-}\left({\bar{A}}^{I}\right)=\Delta^{I}\setminus \left\{a^{I}\right\}$ ($\neq \Delta^{I}$).

As a result, we have the following corollary.

**Corollary 1** For any conflict-free extended concept *C* and any individual *a*,

$$\begin{array}{c} \emptyset {⊨}_{m_{c}}C⊔\neg C\left(a\right). \end{array}$$ (2)

The corollary directly follows Proposition 13 and the definition of conflict-free concept.

## 4 Minimally inconsistent description logic (MIDL)

The basic idea of *minimally inconsistent reasoning* is, by employing a preference relation on models, to select only those preferred models, which contains less inconsistent information, so that the resulting reasoning is more reasonable [38]. We first present a formulation of minimally inconsistent reasoning in MVDL and then investigate its properties and relations.

### 4.1 Minimally inconsistent semantics

Because the constructor of the negation of a role ¬*R* is absent in ALC (see [21]), the inconsistency of KBs is mainly caused by concepts. To convey our core idea, we will define a preference relation according to concepts. For expressive DLs with ¬*R*, we can feasibly extend the preference relation on roles.

Firstly, let Σ be a set of concept names and role names. We introduce a preference relation ≼ between two base interpretations w.r.t. Σ.

**Definition 3** Let $I_{1}$ and $I_{2}$ be two base interpretations. We say $I_{1}$ is *more consistent* than $I_{2}$ w.r.t. Σ, denoted $I_{1}{≼}_{\Sigma }I_{2}$, if the followings hold:

1. $\Delta^{I_{1}}=\Delta^{I_{2}}$, i.e., $I_{1}$ and $I_{2}$ have the same domain Δ;
2. ${\text{p}}^{+}\left(A^{I_{1}}\right)\cap {\text{p}}^{-}\left(A^{I_{1}}\right)\subseteq {\text{p}}^{+}\left(A^{I_{2}}\right)\cap {\text{p}}^{-}\left(A^{I_{2}}\right)$ for any concept name *A* ∈ Σ.

We denote $I_{1}{≺}_{\Sigma }I_{2}$ if $I_{1}{≼}_{\Sigma }I_{2}$ but $I_{2}{⋠}_{\Sigma }I_{1}$.

Intuitively, the first condition states that if $I_{1}$ and $I_{2}$ do not share a common domain, then they are incomparable; the second condition ensures that if $I_{1}{≺}_{\Sigma }I_{2}$ then $I_{1}$ contains less inconsistencies than $I_{2}$.

For instance, let Δ = {*a*, *b*} be a domain and Σ = {*A*}. Assume that $I_{1}$ and $I_{2}$ are two base interpretations such that $\Delta =\Delta^{I_{1}}=\Delta^{I_{2}}$, $A^{I_{1}}=\langle \left\{a^{I_{1}}\right\},\left\{b^{I_{1}}\right\}\rangle$ and $A^{I_{2}}=\langle \left\{a^{I_{2}},b^{I_{2}}\right\},\left\{b^{I_{2}}\right\}\rangle$. Then we can easily see $I_{1}{≺}_{\Sigma }I_{2}$. Let $I_{3}$ be a base interpretation such that $A^{I_{3}}=\langle \left\{b^{I}\right\},\left\{a^{I}\right\}\rangle$. Then $I_{1}{⊀}_{\Sigma }I_{3}$ and $I_{3}{⊀}_{\Sigma }I_{1}$.

Moreover, ≺ is *anti-reflexive*, *anti-symmetric* and *transitive*. Formally, for any base interpretations $I_{i}$ (*i* = 1, 2, 3), (1) $I_{1}{⊀}_{\Sigma }I_{2}$; (2) if $I_{1}{≺}_{\Sigma }I_{2}$ then $I_{2}{⊀}_{\Sigma }I_{1}$; and (3) if $I_{1}{≺}_{\Sigma }I_{2}$ and $I_{2}{≺}_{\Sigma }I_{3}$ then $I_{1}{≺}_{\Sigma }I_{3}$.

**Definition 4** Let Σ be a set of concept names and role names and S a set of base interpretations. A base interpretation I in S is *minimal* if there exists no interpretation $I^{\prime }$ in S such that $I^{\prime }{≺}_{\Sigma }I$. Let $\min_{{≺}_{\Sigma }}\left(S\right)$ denote the set of all minimal interpretations in S w.r.t. ≺_Σ_.

Now, we are ready to define a notion of *minimally multi-valued model*.

**Definition 5** Let K be an extended KB. Let Σ be a set of all concept names and role names occurring in K. We define *minimally multi-valued models* as follows: $Mod_{m}^{min}\left(K\right)=\min_{{≺}_{\Sigma }}\left(Mod_{m}\left(K\right)\right)$.

Intuitively, minimally multi-valued models are those multi-valued models which contain minimal numbers of inconsistencies.

For instance, in Example 1, assume that Σ = {*Fly*, *HasWing*, *Fish*, *Piscivore*, *AbnBird*, *Eat*}. We have $I_{2}{≺}_{\Sigma }I_{1}$ since $\emptyset ={\text{p}}^{+}\left({\text{Fly}}^{I_{2}}\right)\cap {\text{p}}^{-}\left({\text{Fly}}^{I_{2}}\right){≺}_{\Sigma }{\text{p}}^{+}\left({\text{Fly}}^{I_{1}}\right)\cap {\text{p}}^{-}\left({\text{Fly}}^{I_{1}}\right)=\left\{{\text{tweety}}^{I_{1}}\right\}$. It can be also verified that $I_{2}$ is a minimally multi-valued model of $K_{a}$ with respect to Δ.

Those multi-valued models with minimal inconsistencies can characterize their semantics in a rational way. For instance, in the KB $A_{a}$ presented in the Introduction, $I_{2}$, stating *tweety* is not an abnormal bird, is a minimal multi-valued model of $A_{a}$ while $I_{1}$, stating *tweety* is an abnormal bird, is not a minimal multi-valued model of $A_{a}$. Because we know that *tweety* can fly and *tweety* is abnormal or can fly, it is widely accepted that *tweety* is not an abnormal bird. In this sense, the minimal model represents more reasonable knowledge.

By Definition 4 and Definition 5, we conclude that $Mod_{m}^{min}\left(K\right)\subseteq Mod_{m}\left(K\right)$ for all KB K. Based on minimally multi-valued models, we can define the corresponding entailment relation between two extended KBs.

**Definition 6** Let $K_{1},K_{2}$ be two extended KBs. We define *minimally multi-valued entailment* as follows: $K_{1}{⊨}_{m}^{min}K_{2}$ if $Mod_{m}^{min}\left(K_{1}\right)\subseteq Mod_{m}\left(K_{2}\right)$.

In general, the minimally multi-valued entailment is a super set of the multi-valued entailment between two extended KBs since each minimally multi-valued model of a KB is a multi-valued model of the KB.

Analogously, we also define *minimally complete multi-valued models* as those models among all complete multi-valued models with the minimum under ≺_Σ_. The *minimally complete multi-valued entailment*, denoted by ${⊨}_{m_{c}}^{min}$, formally, $K{⊨}_{m_{c}}^{min}K^{\prime }$ if $Mod_{m_{c}}^{min}\left(K\right)\subseteq Mod_{m_{c}}\left(K^{\prime }\right)$ where $Mod_{m_{c}}^{min}\left(K\right)$ denotes the set of all minimally complete multi-valued models of K.

The minimally multi-valued entailment ${⊨}_{m}^{min}$ and the minimally complete multi-valued entailment ${⊨}_{m_{c}}^{min}$ are called *minimally inconsistent entailment*, denoted by ⊨^*min*^.

Because the minimally inconsistent entailments focus on those multi-valued models in which inconsistency is minimized, they are between the classical entailment ⊨ and the multi-valued entailments (⊨_*m*_, ⊨_*m*_*c*__).

### 4.2 Properties of MIDL

In this subsection, we enumerate several useful properties of MIDL and several interesting relations with MVDL.

Firstly, we discuss the paraconsistency of MIDL.

**Lemma 3** Let K be a KB. $Mod_{m}^{min}\left(K\right)\neq \emptyset$.

By Lemma 3, minimally inconsistent entailments preserve the paraconsistency of multi-valued entailments.

**Proposition 14** For any KB K, there exists some axiom *φ* such that $K{⊭}^{min}\varphi$.

Proposition 14 directly follows Lemma 3 if let *φ* = ⊥(*a*).

Moreover, the minimally inconsistent DL do not satisfy the monotonic feature.

**Proposition 15** Minimally inconsistent DL is non-monotonic.

For instance, {¬*A*(*a*), *A*⊔*B*(*a*)} ⊭^*min*^ *B*(*a*) while {*A*(*a*), ¬*A*(*a*), *A* ⊔ *B*(*a*)} ⊭^*min*^ *B*(*a*), ⊨^*min*^ is non-monotonic. So the non-monotonic feature of the minimally inconsistent DL makes their inference more reasonable for real world applications.

Our new semantics satisfies another important property, called *consistency-protected*, for paraconsistent reasoning. It ensures that the new entailment is identical to the classical one. Formally, a (paraconsistent) entailment relation ⊨_*p*_ is consistency-protected if, for any two KBs $K_{1}$ and $K_{2}$ with $K_{1}$ being consistent, then $K_{1}{⊨}_{p}K_{2}$ if and only if $K_{1}⊨K_{2}$.

**Proposition 16** ${⊨}_{m_{c}}^{min}$ is consistency-protected.

This proposition easily follows from the fact that every minimally complete multi-valued model of a classical consistent KB can be one-by-one mapped to classical model of KB:

**Proposition 17** Let K be a KB and I a minimally complete multi-valued model of K. If K is consistent then for any concept *C*,

$${\text{p}}^{+}\left(C^{I}\right)\cap {\text{p}}^{-}\left(C^{I}\right)=\emptyset \text{ and }{\text{p}}^{+}\left(C^{I}\right)\cup {\text{p}}^{-}\left(C^{I}\right)=\Delta^{I}.$$

However, ⊨_*m*_, ${⊨}_{m}^{min}$, and ⊨_*m*_*c*__ are not consistency-protected. For instance, ∅ ⊭_*m*_ *A* ⊔ ¬*A*(*a*), $\emptyset {⊭}_{m}^{min}A⊔\neg A\left(a\right)$, and {*A*(*a*), ¬*A* ⊔ *B*(*a*)} ⊭_*m*_*c*__ *B*(*a*).

By Proposition 17, the property of resolution is valid in minimally inconsistent DL for the case of consistent KBs, while it fails in the general case (possibly inconsistent KBs).

For instance, {*A*(*a*), ¬*A*(*a*), *A* ⊔ *B*(*a*)}⊭^*min*^ *B*(*a*) because there is a conflict between *A*(*a*) and ¬*A*(*a*), so the resolution between ¬*A*(*a*) and *A* ⊔ *B*(*a*) is blocked.

In fact, the entailment is a relation between sets of KBs. In this sense, given two entailments ⊨_*x*_ and ⊨_*y*_, we denote ⊨_*x*_ ⊂ ⊨_*y*_ if for any $K,K^{\prime }$, $K{⊨}_{x}K^{\prime }$ implies $K{⊨}_{y}K^{\prime }$ and there exists some $K^{\prime \prime }$ and $K^{\prime \prime \prime }$ such that $K^{\prime \prime }{⊨}_{y}K^{\prime \prime \prime }$ but $K^{\prime \prime }{⊭}_{x}K^{\prime \prime \prime }$.

**Proposition 18** The three followings hold.

1. ⊨_*m*_ ⊂ ⊨_*m*_*c*__;
2. ${⊨}_{m}\;\subset \;{⊨}_{m}^{min}$; and
3. ${⊨}_{m_{c}}\;\subset \;{⊨}_{m_{c}}^{min}$.

However, ${⊨}_{m}^{min}$ and ⊨_*m*_*c*__ are incomparable.

For instance, let A= {¬*A*(*a*), *A*(*a*), *A*⊔*B*(*a*)}. We have $A{⊨}_{m}^{min}B\left(a\right)$ while $A{⊭}_{m_{c}}A^{\prime }⊔\neg A^{\prime }\left(a\right)$. However, $A{⊨}_{m_{c}}A^{\prime }⊔\neg A^{\prime }\left(a\right)$ while $A{⊭}_{m}^{min}B\left(a\right)$.

**Fig 1:**
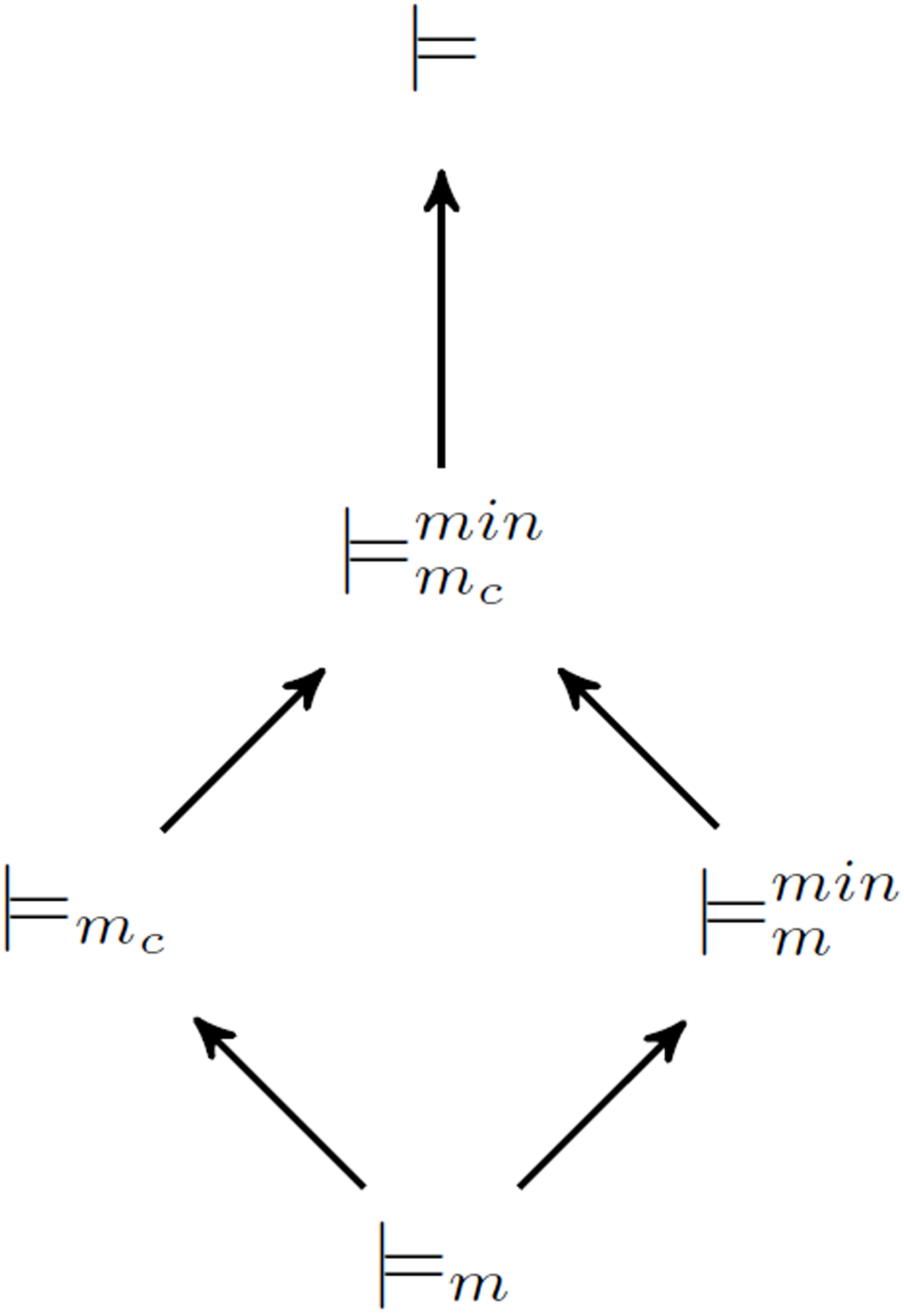
Relations among five entailments: ⊨, ${⊨}_{m},{⊨}_{m_{c}},{⊨}_{m}^{min},{⊨}_{m_{c}}^{min}$.

Based on the discussion above, their relations among four entailments (⊨_*m*_, ⊨_*m*_*c*__, ${⊨}_{m}^{min}$ and ${⊨}_{m_{c}}^{min}$) and classical entailment ⊨ can be shown in Fig 1 where → denotes ⊂.

A more detailed comparison between multi-valued entailment and minimally inconsistent entailment for three inclusions can be shown in Table 2.

**Table 2:** Comparisons for three inclusions under ${⊨}_{m},{⊨}_{m_{c}},{⊨}_{m}^{min},{⊨}_{m_{c}}^{min}$.

Property	↦				⊏				→			
	⊨_*m*_	⊨_*m*_*c*__	${⊨}_{m}^{min}$	${⊨}_{m_{c}}^{min}$	⊨_*m*_	⊨_*m*_*c*__	${⊨}_{m}^{min}$	${⊨}_{m_{c}}^{min}$	⊨_*m*_	⊨_*m*_*c*__	${⊨}_{m}^{min}$	${⊨}_{m_{c}}^{min}$
modus ponens (**MP**)	no	no	yes	yes	yes	yes	yes	yes	yes	yes	yes	yes
modus tollens (**MT**)	no	no	yes	yes	no	no	yes	yes	yes	yes	yes	yes
disjunctive syllogism (**DS**)	no	no	yes	yes	no	no	yes	yes	no	no	yes	yes
resolution (**R**)	no	no	yes	yes	no	no	yes	yes	no	no	yes	yes
disjunctive introduction (**DI**)	yes	yes	yes	yes	yes	yes	yes	yes	yes	yes	yes	yes
implication (**I**)	no	no	no	no	no	no	no	no	no	no	no	no
transitive inclusion (**TI**)	no	no	no	yes	no	no	no	yes	no	no	no	yes
transitivity (**T**)	yes	yes	no	no	yes	yes	no	no	yes	yes	no	no
excluded Middle (**EM**)	no	yes	no	yes	no	yes	no	yes	no	yes	no	yes
monotonicity (**M**)	yes	yes	no	no	yes	yes	no	no	yes	yes	no	no

### 4.3 Usage of MIDL

In Section 3 and Section 4, we introduce four semantics and investigate some properties under three kinds of inclusions. Since our proposal consists of 12 cases which are slightly different shown in Table 2, we are interesting to discuss the relations among them so that we could choose a suitable sematics.

Next, we will classify those relations in three classes.

1. ⊨_*m*_ **vs**. ⊨_*m*_*c*__ **and** ${⊨}_{m}^{min}$ **vs**. ${⊨}_{m_{c}}^{min}$ The difference between ⊨_*m*_ and ⊨_*m*_*c*__ is the law of excluded middle (**EM**) under three kinds of inclusions as well as the difference between ${⊨}_{m}^{min}$ and ${⊨}_{m_{c}}^{min}$. The law of excluded middle can represent tautologies well, such as ⊤ ⊑ ⊥, ⊥(*a*), and *A* ⊓ ¬*A*(*a*). Both ⊨_*m*_*c*__ and ${⊨}_{m_{c}}^{min}$ can satisfy the law of excluded middle while neither ⊨_*m*_ nor ${⊨}_{m}^{min}$ does so. In this sense, we recommend ⊨_*m*_*c*__ or ${⊨}_{m_{c}}^{min}$ if a tautology should be inferred in a scenario. Otherwise, ⊨_*m*_ and ${⊨}_{m}^{min}$ look simple in usage for those users who assume that tautologies do not bring much valuable information.
2. ⊨_*m*_ **vs**. ${⊨}_{m}^{min}$ **and** ⊨_*m*_*c*__ **vs**. ${⊨}_{m_{c}}^{min}$ The differences between ⊨_*m*_ vs. ${⊨}_{m}^{min}$ are mainly the four principles, namely, disjunctive syllogism (**DS**), resolution (**R**), transitivity (**T**), and monotonicity (**M**) under three kinds of inclusions as well as the differences between ⊨_*m*_*c*__ and ${⊨}_{m_{c}}^{min}$. The first two principles (**DS** and **R**) can enhance the inference power by inferring a new conclusion implied by two assertions containing complementary knowledge and the last two principles (**T** and **M**) can characterize the transitivity of inference via subsumption of knowledge bases. Both ${⊨}_{m}^{min}$ and ${⊨}_{m_{c}}^{min}$ can satisfy the four principles while neither ⊨_*m*_ nor ⊨_*m*_*c*__ does so. In other words, both ${⊨}_{m}^{min}$ and ${⊨}_{m_{c}}^{min}$ are non-monotone while ⊨_*m*_ nor ⊨_*m*_*c*__ are monotone. In this sense, we recommend ${⊨}_{m}^{min}$ or ${⊨}_{m_{c}}^{min}$ if the strong inference power is required in a scenario. Otherwise, ⊨_*m*_ and ⊨_*m*_*c*__ are good choices if the monotonicity of inference is guaranteed in some scenario.
3. ↦ **vs**. ⊏ **vs**. → The differences among ↦, ⊏, and → are mainly the three principles related to inclusion, namely, modus ponens (**MP**), modus tollens (**MT**), and transitive inclusion (**DI**) (except ${⊨}_{m_{c}}^{min}$ under →). **MP** characterizes *forward reasoning* while **MT** captures *backward reasoning* [1, 45]. All ⊨_*m*_, ⊨_*m*_*c*__, ${⊨}_{m}^{min}$, and ${⊨}_{m_{c}}^{min}$ under → can satisfy both **MP** and **MT**. All ⊨_*m*_, ⊨_*m*_*c*__, ${⊨}_{m}^{min}$, and ${⊨}_{m_{c}}^{min}$ under ⊏ can satisfy **MP**. Moreover, both ${⊨}_{m}^{min}$ and ${⊨}_{m_{c}}^{min}$ under ⊏ can still satisfy **MT**. Both ${⊨}_{m}^{min}$ and ${⊨}_{m_{c}}^{min}$ can satisfy **MP** while neither ⊨_*m*_ nor ⊨_*m*_*c*__ does so. In a short, ↦ has a stronger inference power than ⊏ and ⊏ has a stronger inference power than →. Of course, → has more cost of reasoning than ⊏ and ⊏ has more cost of reasoning than ↦. The choice of three kinds of inclusion deponds on the requirement of a practical application.

### 4.4 A practical example

Consider a simplified buggy Policy KB [27] $K_{p}=\left(T_{p},A_{p}\right)$ with $T_{p}=\left\{\varphi_{1},\ldots ,\varphi_{5}\right\}$ and $A_{p}=\left\{\varphi_{6}\right\}$ as follows:

$$\begin{array}{c} \begin{array}{cccc} \varphi_{1}: & \text{GeneralReliabilityUserPolicy} & ⊑ & \text{Reliable};\\ \varphi_{2}: & \text{GeneralReliabilityUserPolicy} & ⊑ & \text{Policy};\\ \varphi_{3}: & \text{GeneralReliabilityUserPolicy} & ⊑ & \neg \text{Messaging};\\ \varphi_{4}: & \text{Kerberos} & ⊑ & \neg \text{Messaging};\\ \varphi_{5}: & \text{Reliable} & ⊑ & \text{Messaging};\\ \varphi_{6}: & \text{GeneralReliabilityUserPolicy}(\text{id}). \end{array} \end{array}$$

In $K_{p}$, from *GeneralReliabilityUserPolicy* ⊑ *Messaging* which can be inferred from *φ*_3_ and *φ*_10_ (i.e., *GeneralReliabilityUserPolicy* is of *Messaging*), together with *φ*_5_ (i.e., *GeneralReliabilityUserPolicy* is not of *Messaging*), *GeneralReliabilityUserPolicy* is an unsatisfiable concept. However, *φ*_19_ states that *id* is an instance of the concept *GeneralReliabilityUserPolicy*. As a result, $K_{p}$ is inconsistent.

Next, we compare the four semantics defined above with the following queries *α*_*i*_:

$$\begin{array}{ll} \alpha_{1}=Reliable(id); & \alpha_{2}=Message(id);\\ \alpha_{3}=Kerberos⊔\neg Kerberos(id); & \alpha_{4}=\neg Kerberos(id). \end{array}$$

Intuitively, *α*_1_, *α*_2_, *α*_3_, and *α*_4_ say that *id* is reliable; *id* is a message; *id* either belongs to *Kerberos* or does not; and *α*; and *id* does not belong to *Kerberos*, respectively.

The answer to *α*_*i*_ (*i* = 1, 2, 3, 4) over $K_{p}$ is shown in Table 3.

**Table 3:** Querying over $K_{p}$ under ${⊨}_{m},{⊨}_{m}^{min},{⊨}_{m_{c}},{⊨}_{m_{c}}^{min}$.

*α*_1_	⊨_*m*_	${⊨}_{m}^{min}$	⊨_*m*_*c*__	${⊨}_{m_{c}}^{min}$	*α*_2_	⊨_*m*_	${⊨}_{m}^{min}$	⊨_*m*_*c*__	${⊨}_{m_{c}}^{min}$
↦	no	no	no	no	↦	no	no	no	no
⊏	yes	yes	yes	yes	⊏	yes	yes	yes	yes
→	yes	yes	yes	yes	→	yes	yes	yes	yes
*α*_3_	⊨_*m*_	${⊨}_{m}^{min}$	⊨_*m*_*c*__	${⊨}_{m_{c}}^{min}$	*α*_4_	⊨_*m*_	${⊨}_{m}^{min}$	⊨_*m*_*c*__	${⊨}_{m_{c}}^{min}$
↦	no	no	yes	yes	↦	no	no	no	no
⊏	no	no	yes	yes	⊏	no	no	no	no
→	no	no	yes	yes	→	no	no	no	no

Based on Table 3, the answers to *α*_*i*_ (*i* = 1, 2, 4) with three kinds of inclusions are identical under different semantics. However, the answer to *α*_3_ cannot be inferred in ⊨_*m*_ or ${⊨}_{m}^{min}$, that is to say, we cannot conclude that *id* either belongs to *Kerberos* or does not under both multi-valued semantics and minimally multi-valued semantics, but positive under ⊨_*m*_*c*__ and ${⊨}_{m_{c}}^{min}$, that is to say, we can conclude that *id* either belongs to *Kerberos* or does not under both complete multi-valued semantics and minimally complete multi-valued semantics. In other words, ⊨_*m*_*c*__ and ${⊨}_{m_{c}}^{min}$ look more reasonable in handling the query *α*_3_ since *α*_3_ is a tautology. Besides, the answer to *α*_4_ is negative under all semantics, that is to say, we can always conclude that *id* does not belong to *Kerberos* under all four semantics. Indeed, there is no knowledge about *id* being in *Kerberos* or not. In this sense, all semantics are reasonable in handling *α*_4_.

Now, we add a new assertion *φ*_20_ = ¬(*Reliable* ⊓ *Kerberos*)(*id*) (i.e., *id* is neither *reliable* nor in *Kerberos*) in $A_{p}$ and denote the new KB as $K_{p}^{\prime }$.

The answers to four queries over $K_{p}^{\prime }$ are shown in Table 4.

**Table 4:** Querying over $K_{p}^{\prime }$ under ${⊨}_{m},{⊨}_{m}^{min},{⊨}_{m_{c}},{⊨}_{m_{c}}^{min}$.

*α*_1_	⊨_*m*_	${⊨}_{m}^{min}$	⊨_*m*_*c*__	${⊨}_{m_{c}}^{min}$	*α*_2_	⊨_*m*_	${⊨}_{m}^{min}$	⊨_*m*_*c*__	${⊨}_{m_{c}}^{min}$
↦	no	yes	no	yes	↦	no	no	no	no
⊏	yes	yes	yes	yes	⊏	yes	yes	yes	yes
→	yes	yes	yes	yes	→	yes	yes	yes	yes
*α*_3_	⊨_*m*_	${⊨}_{m}^{min}$	⊨_*m*_*c*__	${⊨}_{m_{c}}^{min}$	*α*_4_	⊨_*m*_	${⊨}_{m}^{min}$	⊨_*m*_*c*__	${⊨}_{m_{c}}^{min}$
↦	no	no	yes	yes	↦	no	no	no	no
⊏	no	no	yes	yes	⊏	no	yes	no	yes
→	no	no	yes	yes	→	no	no	no	no

Based on Tables 3 and 4, the answer to *α*_*i*_ (*i* = 1, 2, 3, 4) with three kinds of inclusions is identical under ⊨_*m*_ and ⊨_*m*_*c*__ respectively. However, both the answer to *α*_1_ under the material inclusion and the answer to *α*_4_ under the internal inclusion change under ${⊨}_{m}^{min}$ and ${⊨}_{m_{c}}^{min}$, where the answers to both *α*_1_ and *α*_4_ become positive under the material inclusion and the internal inclusion of minimally inconsistenct semantics respectively. That is to say, we can conclude that *id* is reliable and *id* does not belong to *Kerberos* under those scenarios. Besides, the answer of *α*_4_ over $K_{p}^{\prime }$ is different from the answer of *α*_4_ over $K_{p}$, where, as discussed above, the answer to *α*_4_ is positive under the internal inclusion of minimally inconsistent semantics. It shows that ${⊨}_{m}^{min}$ and ${⊨}_{m_{c}}^{min}$ are more reasonable since there is more knowledge about *id* related to *Kerberos* in $K_{p}^{\prime }$.

## 5 Tableaux for MVDL and MIDL

Tableau-based algorithms (simply, *tableaux*) are popular algorithms for reasoning in DLs and have been implemented in many popular DL reasoners. For instance, FaCT++ (http://owl.man.ac.uk/factplusplus/), Pellet (http://clarkparsia.com/pellet/). In this section, we first develop a framework of tableau-based algorithms for MVDL and complete MVDL, and then expand it into dealing with the minimally inconsistent DL. Tableau-based algorithms take advantage of the finite model property of DL [1] where the consistency of a KB is captured in some finite forest-based data structures.

### 5.1 Tableaux for MVDL

Analogous to tableaux [1], we describe a kind of tableaux for our new DLs, called *multi-valued tableaux*, in the following three steps:

1. Introducing a new negation normal form, called *extended negation normal form, ENNF*, to handle the complement of concepts.
2. Specifying expansion rules since new expansion rules are needed to capture three inclusions (namely, material inclusion, internal inclusion and strong inclusion);
3. Presenting new closeness condition to characterize our tableaux.

We first define the ENNF of MVDL concepts. A concept *C* is in ENNF, if the complement only occurs over a concept name, and the negation only occurs in front of concept names or its complement. For instance, *A*, ¬*A*, $\bar{A}$, $\neg \bar{A}$, $\forall R.\neg \bar{A}$, and $\exists R.(\neg A⊓\bar{B})$ are all in ENNF. However, neither $\bar{\neg A}$ nor $\exists R.\bar{\neg A}$ is in ENNF.

Proposition 1 ensures that each concept can be transformed into an equivalent ENNF concept by pushing complement and negation inwards.

For a concept *C* we denote the ENNF of ¬*C* by $\dot{\neg }C$ and the ENNF of $\bar{C}$ by ∼*C*. Let *clos*(*C*) denote the smallest set that contains *C* and is closed under sub-concepts, $\dot{\neg }$ and ∼. Let K be an extended KB. We use clos(K) to denote the union of the closure of each concept *C* occurring in K. It is not hard to show that the size of clos(K) is polynomial in the size of K.

Secondly, our multi-valued tableaux work on forest data structure where each node *x* is with labeled as L(x), a subset of clos(K) that contains concepts satisfying individual *x*, and each edge is labelled with L(x,y), a subset of *N*_*R*_ that contains role names satisfying two individuals *x*, *y*.

A node *y* is called an *R-successor* of *x* (or *x* an *R*-*predecessor* of *y*) if for some *R*′ with *R*′
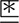
*R* (where 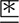 is the transitive closure of ⊑ over roles [1]), *y* is a successor of *x* and $R^{\prime }\in L\left(\langle x,y\rangle \right)$. Similarly, *R*-*neighbors* and *R*-*ancestors* are defined in the standard way.

When the TBox is empty, the multi-valued tableaux would always terminate. However, the multi-valued tableaux for a KB (eg. a KB consisting of a cyclic TBox [1]) would not always terminate similar to the standard tableaux. Our proposed tableaux also employ a so-called blocking technique [1] to ensure termination and correctness.

Next, we briefly recall the blocking technique.

A node *y* is an ancestor of a node *x* if they both belong to a same completion tree and either *y* is a predecessor of *x*, or there exists a predecessor *z* of *x* such that *y* is an ancestor of *z*. A node *x* is *blocked* if there is an ancestor *y* of *x* such that L(x)⊆L(y) (in this case we say that *y* blocks *x*), or if there is an ancestor *z* of *x* such that *z* is blocked.

Thirdly, our expansion rules (shown in Table 5) enrich the standard rules [1] by adding three rules (↦-rule, ⊏-rule and →-rule) for three inclusions (*C* ↦ *D*, *C* ⊏ *D*, and *C* → *D*) in MVDL respectively.

**Table 5:** Multi-valued expansion rules.

⊓-rule	If:	1. $C_{1}⊓C_{2}\in L\left(x\right)$, *x* is not blocked, and
		2. $\left\{C_{1},C_{2}\right\}⊈L\left(x\right)$.
	Then:	set $L\left(x\right):=L\left(x\right)\cup \left\{C_{1},C_{2}\right\}$.
⊔-rule	If:	1. $C_{1}⊔C_{2}\in L\left(x\right)$, *x* is not blocked, and
		2. $\left\{C_{1},C_{2}\right\}\cap L\left(x\right)=\emptyset$.
	Then:	set L(x):=L(x)∪{C} for some *C* ∈ {*C*_1_, *C*_2_}.
∃-rule	If:	1. ∃R.C∈L(x), *x* is not blocked, and
		2. *x* has no *R*-successor *y* with C∈L(y).
	Then:	create a new node *y* with L(x,y):={R}, and L(y):={C}.
∀-rule	If:	1. ∀R.C∈L(x) and R∈L(x,y), *x* is not blocked, and
		2. there is an *R*-successor *y* of *x* with C∉L(y).
	Then:	set L(y):=L(y)∪{C}.
↦-rule	If:	1. C↦D∈T, and
		2. $\dot{\neg }C⊔D\notin L\left(y\right)$.
	Then:	set $L\left(y\right):=L\left(y\right)\cup \left\{\dot{\neg }C⊔D\right\}$.
⊏-rule	If:	1. C ⊏ D∈T, and
		2. ∼C⊔D∉L(y).
	Then:	set L(y):=L(y)∪{∼C⊔D}.
→-rule	If:	1. C→D∈T, and
		2. $\left\{∼C⊔D,\dot{\neg }C⊔\dot{\neg }\bar{D}\right\}⊈L\left(y\right)$.
	Then:	set $L\left(y\right):=L\left(y\right)\cup \left\{∼C⊔D,\dot{\neg }C⊔\dot{\neg }\bar{D}\right\}$.

Finally, we consider which clashes over concept names can characterize our close conditions of a complete forest.

The clash {*A*, ¬*A*} of the standard tableau can be classified as three kinds of clashes over concept names as follows:

$$\begin{array}{ccc} C1:\;\left\{A,\;\neg A\right\}; & C2:\;\left\{A,\;\bar{A}\right\}\text{ or }\left\{\neg A,\;\neg \bar{A}\right\}; & C3:\;\left\{\bar{A},\;\neg \bar{A}\right\}. \end{array}$$

Intuitively speaking,

1. the **C1** clash is used to capture classical inconsistency;
2. the **C2** clash is used to capture multi-valued inconsistency;
3. the **C3** clash is used to capture complete multi-valued inconsistency.

The multi-valued tableaux initializes a forest $F_{K}$ consisting only of root nodes. More precisely, $F_{K}$ contains a root node *x*_*a*_ for each individual $a\in N_{I}\left(K\right)$ (i.e., the collection of all individuals occurring in K), and an edge 〈*x*_*a*_, *y*_*b*_〉 if L contains an assertion (*a*, *b*):*R*.

The labels of these nodes and edges are initialized as follows:

$$\{\begin{array}{lll} L\left(x_{a}\right) & ≔ & \left\{C\in clos\left(A\right)|a:C\in A\right\};\\ L\left(\langle x_{a},y_{b}\rangle \right) & ≔ & \left\{R\in N_{R}|\left(a,b\right):R\in A\right\}. \end{array}$$ (3)

Then $F_{K}$ is expanded by repeatedly applying multi-valued expansion rules. We say a forest as a *completion forest* if, and only if no more rules can be applied on it. Let forests(K) denote the collection of all possible completion forests generated by applying multi-valued expansion rules.

Based on such two kinds of clashes, we define two kinds of closedness conditions:

- A forest F is *m*_1_-*closed* if it contains at least a node containing a **C2** clash; and *m*_1_-*open* otherwise;
- A forest F is *m*_2_-*closed* if it contains at least a node containing either a **C2** clash or a **C3** clash; and *m*_2_-*open* otherwise.

Clearly, a *m*_1_-closed forest is *m*_2_-closed.

When a tableau algorithm for K terminates, if it leads to a *m*_1_-open completion forest in the end, then we say “K is multi-valued consistent”; and if it leads to a *m*_2_-open completion forest in the end, then we say “K is complete multi-valued consistent”.

Next result states that our proposal multi-valued tableaux are sound and complete for MVDL and the complete MVDL.

**Theorem 1** Let K=(T,A) be an extended KB. Then

1. K is multi-valued inconsistent if and only if forests(K) contains no *m*_1_-open completion forest;
2. K is complete multi-valued inconsistent if and only if forests(K) contains no *m*_2_-open completion forest.

### 5.2 Tableaux for minimally inconsistent entailment problem

To develop a tableau algorithm for the minimally inconsistent entailment, we introduce a preference relation on completion forests to eliminate those forests which contain redundant inconsistencies.

**Definition 7** Let Σ be a set of concept names and role names. Let $F_{1}$ and $F_{2}$ be two completion forests on Σ. We say $F_{1}$ is *less conflicting than* $F_{2}$ w.r.t. Σ, denoted $F_{1}\;{⊴}_{\Sigma }\;F_{2}$, if the followings hold:

1. $nodes\left(F_{1}\right)=nodes\left(F_{2}\right)$, i.e., $F_{1}$ and $F_{2}$ have the same set of nodes;
2. if {A,¬A}⊆L(x) in $F_{1}$ implies {A,¬A}⊆L(x) in $F_{2}$ for any concept name *A* ∈ *N*_*C*_ and for any node *x*.

We denote $F_{1}\;{⊲}_{\Sigma }\;F_{2}$ if $F_{1}\;{⊴}_{\Sigma }\;F_{2}$ but $F_{2}\;{⋬}_{\Sigma }\;F_{1}$.

Intuitively, if $F_{1}$ is less conflicting, than $F_{2}$ then $F_{1}$ contains less **C1** clashes than $F_{2}$ does.

**Definition 8** Let K be an extended KB. We define *minimally conflicting forests* as follows: ${\text{forest}}^{min}\left(K\right)=\min_{{⊲}_{\Sigma }}\text{ forest}\left(K\right)$.

It can be easily verified that there exist always minimally conflicting forests as long as forests exists since completion forests are always finite.

Moreover, there exists a close relation between ≺_Σ_ (defined over (complete) multi-valued models) and ⊲_Σ_ (defined over completion forests).

Let F be a completion forest and I a base interpretation on Δ. We say I *satisfies* F, denoted by I⊨F if the followings hold:

- *x* is in nodes(F) if and only if $x^{I}\in \Delta^{I}$;
- for all node *x*, B∈L(x) if and only if $x^{I}\in B^{I}$ where *B* is in form of $A,\neg A,\bar{A}$;
- for all nodes *x* and *y*, R∈L(x,y) if and only if $\left(x^{I},y^{I}\right)\in R^{I}$.

By the definition, all induced base interpretations from a completion forest satisfy the forest.

We introduce a notion of *induced base interpretation* to connect completion forests with multi-valued models.

**Definition 9** Let F be a completion forest. An *induced base interpretation* $\left(nodes\left(F\right),\cdot^{I}\right)$ from F is a base interpretation where nodes(F) is the collection of all nodes occurring in F and $\cdot^{I}$ maps each individual $a\in N_{I}\left(K\right)$ to a node *x*_*a*_ of F and $\cdot^{I}$ satisfies: for any node *x* and any edge 〈*x*, *y*〉 in F,

1. $x\in {\text{p}}^{+}\left(A^{I}\right)$ if A∈L(x) and $x\in \Delta^{I}\setminus {\text{p}}^{+}\left(A^{I}\right)$ if $\bar{A}\in L\left(x\right)$;
2. $x\in {\text{p}}^{-}\left(A^{I}\right)$ if ¬A∈L(x) and $x\in \Delta^{I}\setminus {\text{p}}^{-}\left(A^{I}\right)$ if $\neg \bar{A}\in L\left(x\right)$;
3. $\left(x,y\right)\in {\text{p}}^{+}\left(R^{I}\right)$ if R∈L(〈x,y〉) and $\left(x,y\right)\in {\text{p}}^{-}\left(R^{I}\right)$ if R∉L(〈x,y〉).

The following result states that the relation ⊲_Σ_ can exactly capture the relation ≺_Σ_.

**Proposition 19** Let Σ be a set of concept names and role names. Let $F_{i}$ be a completion forest on Σ and $I_{i}$ be a base interpretation (*i* = 1, 2) on Σ. If $I_{1}⊨F_{1}$ and $I_{2}⊨F_{2}$ then we conclude that $F_{1}\;{⊴}_{\Sigma }\;F_{2}$ if and only if $I_{1}\;{≼}_{\Sigma }\;I_{2}$.

Next results show that our tableaux restricted to minimally conflicting forests can soundly and completely characterize the reasoning of MIDL.

**Theorem 2** Let K be a KB and *C*, *D* two concepts. The followings hold.

1. $K{⊨}_{m}^{min}C\left(a\right)$ if and only if ${\text{forest}}^{min}(K\cup \left\{\bar{C}\left(a\right)\right\}$ contains no *m*_1_-open completion forest;
2. $K{⊨}_{m}^{min}C\mapsto D$ if and only if ${\text{forest}}^{min}\left(K\cup \left\{\left(\neg \bar{C}⊓\bar{D}\right)\left(\tau \right)\right\}\right)$ contains no *m*_1_-open completion forest;
3. $K{⊨}_{m}^{min}C\;⊏\;D$ if and only if ${\text{forest}}^{min}\left(K\cup \left\{\left(C⊓\bar{D}\right)\left(\tau \right)\right\}\right)$ contains no *m*_1_-open completion forest;
4. $K{⊨}_{m}^{min}C\to D$ if and only if ${\text{forest}}^{min}\left(K\cup \left\{\left(C⊓\bar{D}\right)\left(\tau \right)\right\}\right)$ and ${\text{forest}}^{min}\left(K\cup \left\{\left(\neg D⊓\neg \bar{C}\right)\left(\tau \right)\right\}\right)$ contains no *m*_1_-open completion forest.

**Theorem 3** Let K be a KB and *C*, *D* two concepts. The followings hold.

1. $K{⊨}_{m_{c}}^{min}C\left(a\right)$ if and only if ${\text{forest}}^{min}\left(K\cup \left\{\bar{C}\left(a\right)\right\}\right)$ contains no *m*_2_-open completion forest;
2. $K{⊨}_{m_{c}}^{min}C\mapsto D$ if and only if ${\text{forest}}^{min}\left(K\cup \left\{\left(\neg \bar{C}⊓\bar{D}\right)\left(\tau \right)\right\}\right)$ contains no *m*_2_-open completion forest;
3. $K{⊨}_{m_{c}}^{min}C\;⊏\;D$ if and only if ${\text{forest}}^{min}\left(K\cup \left\{\left(C⊓\bar{D}\right)\left(\tau \right)\right\}\right)$ contains no *m*_2_-open completion forest;
4. $K{⊨}_{m_{c}}^{min}C\to D$ if and only if both ${\text{forest}}^{min}\left(K\cup \left\{\left(C⊓\bar{D}\right)\left(\tau \right)\right\}\right)$ and ${\text{forest}}^{min}\left(K\cup \left\{\left(\neg D⊓\neg \bar{C}\right)\left(\tau \right)\right\}\right)$ contains no *m*_2_-open completion forest.

Next, we use an example to demonstrate our tableaux for MIDL with instance checking.

Let A be an ABox which contains seven assertions *ψ*_*i*_ (*i* = 1, …, 7) shown in Table 6. And consider there are three queries *β*_*i*_ (*i* = 1, 2, 3) as follows:

**Table 6:** Assertions in A.

*ψ*_1_	*OvoVegetarian*(*id*)
*ψ*_2_	*eats*(*id*, *food*)
*ψ*_3_	¬*OvoVegetarian* ⊔ ∀*eats*.*OvoVegetarianFood*(*id*)
*ψ*_4_	¬*OvoVegetarian* ⊔ ∀*eats*.*OvoLactoVegetarianFood*(*id*)
*ψ*_5_	¬*OvoLactoVegetarianFood* ⊔ *LactoVegetarianFood*(*fd*)
*ψ*_6_	¬(*LactoVegetarianFood* ⊓ *OvoVegetarianFood*)(*fd*)
*ψ*_7_	¬*LactoVegetarianFood* ⊔ *Food*(*fd*)

$$\begin{array}{l} \beta_{\text{1}}=OvoVegetarian(id)\text{;}\\ \beta_{\text{2}}=\neg OvoLactoVegetarianFood\;⊔\;OvoVegetarianFood\;⊔\;Food(fd);\\ \beta_{\text{3}}=\neg OvoLactoVegetarianFood\;⊔\;OvoLactoVegetarianFood(fd). \end{array}$$

Initially, the algorithm starts at $F_{K}$ for $A\cup \{\bar{C}\left(a\right)\}$ *C*(*a*) ∈ {*β*_1_, *β*_2_, *β*_3_} and then a completion forest is obtained by exhaustively applying the multi-valued expansion rules. We abbreviate *OvoVegetarian* to *OV*, *LactoVegetarianFood* to *LVF*, *OvoLactoVegetarianFood* to *OLVF* and *OvoVegetarianFood* to *OVF* respectively.

1. To answer *β*_1_, all forests in ${\text{forest}}^{min}\left(K\right)$ are both *m*_1_-closed and *m*_2_-closed.
2. To answer *β*_2_, let F be a forest which contains two nodes:
  - L(id)={OV, ¬*OV*};
  - L(fd)={LVF, ¬*LVF*, ¬*LVF*, $\neg \bar{\text{OLVF}}$,$\bar{\text{OVF}}$, $\bar{\text{Food}}\}$ and an edge L(id,fd)={eats}.
  As a result, $F\in {\text{forest}}^{min}\left(K\right)$ and F is neither *m*_1_-closed nor *m*_2_-closed. However, all minimally completion forests are both *m*_1_-closed and *m*_2_-closed.
3. To answer *β*_3_, let F be a forest which contains two nodes:
  - L(id)={OV, ¬*OV*};
  - L(fd)={LVF, ¬*LVF*, *Food*, $\neg \bar{\text{OLVF}}$, $\bar{\text{OLVF}}\}$ and an edge L(id,fd)={eats}.
  As a result, $F\in {\text{forest}}^{min}\left(K\right)$ and F is not *m*_1_-closed but *m*_2_-closed. Analogously, for any minimally completion forest $F^{min}$, $F^{min}$ is not *m*_1_-closed but *m*_2_-closed.

Finally, the answers of *β*_*i*_ in A are shown in Table 7.

**Table 7:** Querying over A.

Query	⊨_*m*_	${⊨}_{m}^{min}$	⊨_*m*_*c*__	${⊨}_{m_{c}}^{min}$
*β*_1_	yes	yes	yes	yes
*β*_2_	no	no	yes	yes
*β*_3_	no	yes	no	yes

In the end of this section, we discuss the complexity of different semantics proposed in this paper.

**Theorem 4** Let A be an ABox and *C*(*a*) be an assertion in ALC. The problem of deciding if $A{⊨}_{p}C\left(a\right)$ is PSPACE-complete where ⊨_*p*_ ∈ {⊨_*m*_, ⊨_*m*_*c*__, ⊨^*min*^}.

**Theorem 5** Let A be an ABox, T be a TBox and *C*(*a*) be an assertion in ALC. The problem of deciding if $\left(A,T\right){⊨}_{p}C\left(a\right)$ is ExpTime-complete where ⊨_*p*_ ∈ {⊨_*m*_, ⊨_*m*_*c*__, ⊨^*min*^}.

In other words, the complexity of minimally inconsistent entailment is no harder than that of classical entailment in DL.

## 6 Related works

As explained earlier, several paraconsistent DLs and non-monotonic DLs have been proposed to inconsistency handling. In this section we compare our work with some existing paraconsistent DLs and non-monotonic DLs.

**Paraconsistent DL** Compared with existing four-valued semantics and three-valued semantics of DLs, our multi-valued DL provides a framework, in which existing proposals for three-valued [5, 39] and four-valued DLs [21] can be characterized. Besides, PALC in [19], based on the description logic ${\text{ALC}}_{∼}^{n}$ which extends a dual (or multiple)-interpretation semantics for ALC, introduces a paraconsistent negation for tolerating inconsistency similar to the strong negation in Nelson’s paraconsistent logic N4 [31]. In this paraconsistent DL, the paraconsistent negation corresponds to the classical negation while their classical negation corresponds to the complement in our multi-valued DL. In PALC, the satisfaction of GCIs is defined by the internal inclusion. In this sense, PALC is a fragment of multi-valued DL. Our minimally inconsistent DL satisfies some useful properties, such as disjunctive syllogism (DS), resolution and non-monotonicity (see Table 2) which are failed by those paraconsistent DLs based on multi-valued DLs. The quasi-classical DL in [26, 28, 30] is a major variant of paraconsistent DLs, in which two semantics, namely weak semantics and strong semantics, are introduced to characterize quasi-classical semantics so that DS and resolution valid. There are some similarities and differences between minimally inconsistent semantics and quasi-classical semantics.

- (Similarity). They follow four-valued semantics [43] to tolerate (classical) inconsistency, where each concept (or role) is interpreted to a set of instances, the two types of interpretations will map every concept to a pair of sets of instances, where the former characterizes those instances certain to belong to the concept, and the latter characterizes those instances certain not to belong to the concept. Moreover, the (classical) negation is weakened and a new negation (called by “complement” in our semantics and “QC-negation” in quasi-classical semantics) is introduced to characterize “clash” (or “opponent”) in the tableaux which is developed to solve their entailment problems. Additionally, both multi-valued semantics (incl. complete multi-valued semantics) and quasi-classical semantics are monotonic.
- (Difference). Compared to quasi-classical semantics, our minimally inconsistent semantics is planting a non-monotonic feature in reasoning so that conclusions are more reasonable. For instance, let A={¬A(a),A(a), *A* ⊔ *B*(*a*)}, under both quasi-classical semantics and minimally inconsistent semantics, *A*(*a*), ¬*A*(*a*) and *A* ⊔ *B*(*a*) are taken as contradictions. Under quasi-classical semantics, we can draw *B*(*a*) from A while we can not draw *B*(*a*) from A under minimally inconsistent semantics. Because three of them are contradictions, *B*(*a*) could be any of four values following from four-valued logics. In other words, the answer “unknown” to *B*(*a*) looks more reasonable than the answer “yes” to it. Moreover, compared to quasi-classical semantics, our complete multi-valued semantics can capture the law of excluded middle and our minimally complete multi-valued semantics is equal to the classical semantics in the scenario of (classical) consistent knowledge (i.e., the property of consistency-protected). Additionally, compared to the tableau-based reasoning algorithm where a new rule called *QC-rule* is added to enhance the power of reasoning [28], our tableau-based algorithm is redefining the definitions of both clashes and closedness so that meaningless conclusions are rejected and reasonable conclusions are inferred.

Several approaches for paraconsistent DLs are based on repairing [3, 18, 20, 23], in which a new consistent KB or set of models of KB are restored from an inconsistent KB by removing some knowledge causing inconsistency. There are also approaches for paraconsistent DLs based on argumentation presented [24, 46] and based on distances [25, 29]. In argumentation-based approaches, some partial orders (argument principles) of all consistent subsets of an inconsistent KB are introduced so that preferred consistent subsets of the KB are selected. In distance-based approaches, some distances functions are introduced to compute the minimal two-valued interpretations taken as candidate models of a (possibly inconsistent) KB. Our approach adopts a different principle from these approaches in that ours does not reject any knowledge but tolerate inconsistent knowledge in reasoning.

As an important member of the multi-valued DL family, fuzzy description logics [8, 24] can reason with uncertain knowledge in DL. Fuzzy DL admits truth values different from “true” and “false”, each of which is intuitively taken as a certain degree. Usually, the set of possible truth values is the whole interval [0, 1]. Though some properties such as MP, MT and DS are valid in some fuzzy DL, the main difference between fuzzy logic and multi-valued logic is in the aims, where fuzzy logic is based on fuzzy interpretations (a function from a domain to [0, 1]), while multi-valued logic is based on multi-valued interpretations (a function from a domain to a set of discrete values).

**Non-monotonic DL** Three major formalisms of non-monotonic reasoning (default logic [7], autoepistemic logic [12, 13], and circumscription [7, 34]) have been adapted to DLs. The big difference between our minimally inconsistent reasoning and those non-monotonic reasoning in DLs inherits the difference between paraconsistent logic (which deals with contradictions of static KBs) and non-monotonic logic (which handles with contradictions arising from further evidence). A detailed comparison is as follows:

- The power of tolerating inconsistency is different. Our extension maintains the paraconsistency which could be applied to deal with inconsistencies while the ability of those non-monotonic extensions in handling contradictions (where some contradictions are treated as exceptions) is limited. In the default extension of DLs [7], there might not be a default extension of a default KB because of inconsistency [37]. In the autoepistemic extension of DLs [12, 13], inconsistencies between ontologies and rules are hardly tolerated [36]. In the circumscription extension of DL [7, 34], each circumscribed TBox must be consistent so that the partial order ≺_*CP*_ over models of that TBox can be definable.
- The strategy of tolerating inconsistency is different. Our two minimally inconsistent semantics are introducing multi-valued semantics to weaken the negation ¬, so that the inconsistency could be taken as a “part”. However, those current non-monotonic DLs introduce some new “operators” in syntax and within semantics of those operators, the inconsistency is treated as “exception”. In the default extension of DLs [7], open default rules are introduced to express a non-monotonic relation between concepts. In the autoepistemic extension of DLs [12, 13], epistemic operators are defined as new constructors of DLs. In the circumscription extension of DLs [7, 34], concept circumscription patterns *CP* are introduced to limit the extension of concepts by restricting nonname individuals.
- The principle of semantics is different. Our four semantics are all multi-valued while those non-monotonic semantics are two-valued.

Recently, a non-monotonic DL named $\text{ALC}+T_{\text{min}}$ proposes a non-monotonic reasoning about prototypical properties and inheritance with exceptions in DL [15, 33]. In the following, we compare our work to the non-monotonic DL $\text{ALC}+T_{\text{min}}$. Indeed, the differences discussed above between our minimally inconsistent DL and existing non-monotonic DLs also exist between our minimally and $\text{ALC}+T_{\text{min}}$ as follows:

- As discussed in [33], the fact that a KB may have no minimal model leads to a explosive reasoning while our two minimally inconsistent semantics are paraconsistent.
- In syntax, a typicality operator **T** is introduced to capture non-exceptions. Though the complement of a concept is introduced in syntax to characterize the (minimally) multi-valued inconsistency, our four semantics are suitable for classical KBs and all techniques are feasible in classical DLs since classical DL is taken as a subfragment of MVDL.
- Our four semantics are all multi-valued while the semantics of $\text{ALC}+T_{\text{min}}$ is two-valued.

Moreover, though both our minimally inconsistent DL and $\text{ALC}+T_{\text{min}}$ (including DLs with circumscription [10, 34]) introduce some “minimal model” semantics to infer more reasonable/defeasible conclusions by minimizing inconsistent/atypical information, they are based on slightly different strategies. Under our minimally inconsistent semantics, minimal models are selected from multi-valued models while under the semantics of $\text{ALC}+T_{\text{min}}$, minimal models are selected from two-valued models containing less exceptions. As a result, they could bring different conclusions. Let us recall the example presented in [33] as follows:

Let $K_{john}=\left(T_{john},A_{john}\right)$ where the TBox $T_{john}=\{T\left(\text{Athlet}\right)⊑\text{Confident}$, **T**(*Athlet*) ⊓ *Finnish* ⊑ ¬*Confident*} and the ABox $A_{john}=\{\text{Athlet}\left(\text{john}\right)$, *Finnish*(*john*)}. As discussed in [33], **T**(*Athlet*)(*john*) is no longer derivable while it can be still derivable under our minimally inconsistent semantics. Moreover, our minimally inconsistent DL is multi-valued while $\text{ALC}+T_{\text{min}}$ is (classically) two-valued. For instance, *Confident*(*john*) is no longer derivable under the semantics of $\text{ALC}+T_{\text{min}}$. Instead, ¬*Confident*(*john*) can be inferred under the semantics of $\text{ALC}+T_{\text{min}}$. However, under our minimally inconsistent semantics of internal inclusion, we can infer both *Confident*(*john*) and ¬*Confident*(*john*) are true. That is to say, *Confident*(*john*) is a classical “contradiction”. Finally, though both minimally complete multi-valued DL and $\text{ALC}+T_{\text{min}}$ have the same power as DL in treating classical consistent KB, they have slightly different capability in treating inconsistent KB.

Besides, a preferential tableau algorithm in [16] for circumscription in DLs minimizes the extension of concepts while our tableau algorithm minimizes inconsistency. Additionally, compared with argumentative reasoning [27, 46], localizing reasoning in sub-KBs, our approach investigates whole KBs. The argumentative reasoning is too cautious in the sense that a query could be answered only by exhaustedly arguing, thus losing potentially useful conclusions in the argumentative reasoning. In the MKNF extension of DLs [36], two modal operators: **K** and **not** are introduced as new syntax constructors to tolerate inconsistency between ontologies and rules.

In the last of this section, we compare to our previous processings [39, 40]. In previous proceedings, we present a minimally inconsistent semantics [40] based on our proposed paradixcal semantics [39]. In this paper, we propose a multi-valued framework which can produce 12 semantics including the two semantics defined in [40] and four-valued semantics under three kinds of inclusion [21, 43]. In other words, seven kinds of semantics are new. Structurally, Section 3 extends our previous proceedings [39] and some existing properties stated in [21, 43]. Section 4 extends our previous proceedings [40] by adding investigations of seven kinds of new semantics. Section 5 is totally new.

## 7 Conclusions and future works

In this paper, we have proposed a framework for multi-valued paraconsistent DLs. Several major proposals for paraconsistent DLs can be embedded in our framework. We have also introduced a non-monotonic paraconsistent extension of the classical DL, with which inconsistency in reasoning can be minimized. The suitability of the framework and the minimally inconsistent DL is justified by several important properties. In particular, MIDL overcomes some shortcomings of existing paraconsistent DLs and non-monotonic DLs. The paper mainly focuses on the theoretical research of reasoning with inconsistent ontologies based on multi-valued logic. The implementation of this framework is beyond this paper. There are some paraconsistent reasoners with ontologies such as ParOWL [43], PROSE [26, 30], and QC-OWL [47]. In this paper, we have developed a new tableaux for multi-valued DLs and showed that the tableau is sound and complete for the minimally inconsistent DL. Such a tableaux can be taken as a framework for implementing multi-valued DLs and their non-monotonic extensions. The proposed tableau can also be used to develop new tableaux for other non-monotonic DLs. In the future, we plan to implement the proposed algorithm and explore applications of this nonclassical reasoning in ontology management. Meanwhile, we consider the extension of this semantics to expressive DL languages since the finite model property does not hold in some of expressive DL.

## Appendix: Proofs

*Proof of Lemma 2*. Let ⊨_*p*_ be a paraconsistent entailment. Assume that ⊨_*p*_ satisfies DS, DI and transitivity. Given a ABox $A_{1}=\left\{A\left(a\right),\neg A\left(a\right)\right\}$, $A_{1}$ is inconsistent. Because ⊨_*p*_ satisfies DI, $A_{1}{⊨}_{p}A⊔B\left(a\right)$. Let $A_{2}=\left\{A\left(a\right),\neg A\left(a\right),A⊔B\left(a\right)\right\}$. We conclude that $A_{1}{⊨}_{p}A_{2}$. Because ⊨_*p*_ satisfies DS, we conclude that $A_{2}{⊨}_{p}B\left(a\right)$. Then, $A_{1}{⊨}_{p}B\left(a\right)$ since ⊨_*p*_ satisfies transitivity.

*Proof of Proposition 1*. Those claims hold by applying the analogous proofs in [43].

*Proof of Proposition 2*. This claim holds by applying the analogous proofs in [43].

*Proof of Proposition 3*. Given a classical KB K, consider a contradiction ¬*A* ⊓ *A*(*a*) where *A* and *a* do not appear in K, we conclude that $Mod_{m}\left(SF\left(K\right)\right)\neq \emptyset$ and $Mod_{m}\left(SF\left(K\right)\right)\neq \subseteq Mod_{m}\left(SF\left(\left\{\neg A⊓A\left(a\right)\right\}\right)\right)$. Therefore, ¬*A* ⊓ *A*(*a*) is desired.

*Proof of Proposition 4*. Those claims hold by applying the analogous proofs in [21] and [43].

*Proof of Proposition 5*. We need to show that ⊨_*m*_ satisfies the property of monotonicity. That is, let $K_{1},K_{2},K_{3}$ be three extended KBs and $K_{1}\subseteq K_{3}$, $K_{1}{⊨}_{m}K_{2}$ implies $K_{3}{⊨}_{m}K_{2}$. By the definition of ⊨_*m*_, if $K_{1}\subseteq K_{3}$ then $Mod_{m}\left(K_{3}\right)\subseteq Mod_{m}\left(K_{1}\right)$. Because for any KB K whose axioms are enumerated as *φ*_1_, …, *φ*_*n*_, we conclude that $Mod_{m}\left(K\right)=Mod_{m}\left(\left\{\varphi_{1}\right\}\right)\cap \ldots \cap Mod_{m}\left(\left\{\varphi_{n}\right\}\right)$ by Definition 1 and Definition 2. Then *Mod*_*m*_(*φ*) ∩(*Mod*_*m*_({*φ*_1_})∩⋯∩*Mod*_*m*_({*φ*_*n*_}))âŠ†*Mod*_*m*_({*φ*_1_})∩⋯∩*Mod*_*m*_({*φ*_*n*_}) for any *φ*. Therefore, $Mod_{m}\left(K_{3}\right)\subseteq Mod_{m}\left(K_{1}\right)$. Because $K_{1}{⊨}_{m}K_{2}$, $Mod_{m}\left(K_{1}\right)\subseteq Mod_{m}\left(K_{2}\right)$. Then $K_{3}{⊨}_{m}K_{2}$.

*Proof of Proposition 6*.

1. If $T{⊨}_{m}C\;⊏\;D$ and $T{⊨}_{m}D\;⊏\;E$ then for any base interpretation I, $I{⊨}_{m}T$ implies ${\text{p}}^{+}\left(C^{I}\right)\subseteq {\text{p}}^{+}\left(D^{I}\right)$ and ${\text{p}}^{+}\left(D^{I}\right)\subseteq {\text{p}}^{+}\left(E^{I}\right)$. Then ${\text{p}}^{+}\left(C^{I}\right)\subseteq {\text{p}}^{+}\left(E^{I}\right)$, that is, $I{⊨}_{m}C\;⊏\;E$.
2. $T{⊨}_{m}\neg E\;⊏\;\neg D$ and $T{⊨}_{m}\neg D\;⊏\;\neg C$ then $T{⊨}_{m}\neg E\;⊏\;\neg C$ by the proof of (1). It directly follows from (1) since $I{⊨}_{m}C\to D\Leftrightarrow I{⊨}_{m}C\;⊏\;D,I{⊨}_{m}\neg D\;⊏\;\neg C$.

*Proof of Proposition 7*. We prove this proposition in a construction method.

- For any base interpretation I, if $I{⊨}_{m}K$ then there exists some four-valued interpretation $I_{4}$ obtained by restricting I in the language of four-valued DL. Because K contains no complement of any concept. Thus $I_{4}{⊨}_{4}K$.
- For any four-valued interpretation $I_{4}$, if $I_{4}{⊨}_{4}K$ then I is a base interpretation such that $I{⊨}_{m}K$.

*Proof of Proposition 8*. This proposition directly follows the definition of complete base interpretations.

*Proof of Proposition 9*. It directly follows Proposition 2 since K contains no complement of some concept.

*Proof of Proposition 10*. We prove this proposition in a construction method.

- For any base interpretation I, if $I{⊨}_{m_{c}}K$ then there exists some paradoxical interpretation $I_{p}$ obtained by restricting I in the language of paradoxical DL. Because K contains no complement of any concept. Thus $I_{p}{⊨}_{LP}K$.
- For any paradoxical interpretation $I_{p}$, if $I_{p}{⊨}_{LP}K$ then there exists some complete base interpretation I such that $I{⊨}_{m}K$. Since $I_{p}$ is complete, $I{⊨}_{m_{c}}K$.

*Proof of Proposition 11*. It directly follows Proposition 4 if we consider complete base interpretations instead of base interpretations.

*Proof of Proposition 12*. If $T{⊨}_{m_{c}}C\mapsto D$ and $T{⊨}_{m_{c}}D\mapsto E$ then for any base interpretation I, $I{⊨}_{m_{c}}T$ implies $\Delta^{I}\setminus {\text{p}}^{-}\left(C^{I}\right)\subseteq {\text{p}}^{+}\left(D^{I}\right)$ and $\Delta^{I}\setminus {\text{p}}^{-}\left(D^{I}\right)\subseteq {\text{p}}^{+}\left(E^{I}\right)$. Because *D* is conflict-free, ${\text{p}}^{+}\left(D^{I}\right)=\Delta^{I}\setminus {\text{p}}^{-}\left(D^{I}\right)$. Then $\Delta^{I}\setminus {\text{p}}^{-}\left(C^{I}\right)\subseteq {\text{p}}^{+}\left(E^{I}\right)$, that is, $I{⊨}_{m_{c}}C\mapsto E$.

*Proof of Proposition 13*. Let Δ = {*a*, …} be a domain. For any complete base interpretation I on Δ, I is a multi-valued model of the empty KB. Because I is complete, ${\text{p}}^{+}(C^{I}\cup {\text{p}}^{-}\left(C^{I}\right)=\Delta^{I}$. Then $a^{I}\in {\text{p}}^{+}\left({\left(C⊔\neg C\right)}^{I}\right)$. Therefore, $I{⊨}_{m_{c}}C⊔\neg C\left(a\right)$.

*Proof of Lemma 3* Let Σ be a set of all concept names and role names occurring in K (obviously, Σ is finite). We will prove this lemma in two steps:

- For any KB K (in satisfiable form) in ALC, there exists some finite multi-valued model of K. By Proposition 2, $Mod_{m}\left(K\right)\neq \emptyset$, K is multi-valued consistent. In [21, 30, 43], K is four-valued consistent if and only if γ(K) is consistent where *γ* is a transformation function defined as follows: assume that all concepts are in negation normal form (NNF), that is, the negation ¬ only occurs in front of concept names, (we use $\dot{\neg }C$ to denote the NNF of ¬*C* (see Section 5)
  – *γ*(⊤) = ⊤; *γ*(⊥) = ⊥; and *γ*(*R*) = *R* for any role *R*;
  – *γ*(*A*) = *A* and *γ*(¬*A*) = *B* where *B* is a fresh concept name, for any *A*;
  – *γ*(*C*_1_ ⊔ *C*_2_) = *γ*(*C*_1_) ⊔ *γ*(*C*_2_) and *γ*(*C*_1_ ⊓ *C*_2_) = *γ*(*C*_1_) ⊓ *γ*(*C*_2_);
  – *γ*(∀*R*.*C*) = ∀*R*.*γ*(*C*); and *γ*(∃*R*.*C*) = ∃*R*.*γ*(*C*).
  Analogously, we can conclude that K is complete multi-valued consistent if and only if $\gamma \left(K\right)\cup T^{+}$ is consistent where
  $$\begin{array}{c} T^{+}:=\left\{⊤⊑\gamma \left(A\right)⊔\gamma \left(\neg A\right)|\;\text{for}\;\text{all}\;\text{concept}\;\text{name}A\;\text{occurring}\;\text{in}\;K\right\}. \end{array}$$
  (*Note that $T^{+}$ is added to ensure that each complete multi-valued interpretation can be translated into an (classical) interpretation*.) Since ALC has the finite model property [1, 45], for any KB K in ALC, if K is consistent then there exists some finite model of K. Then we can conclude that for any KB K in multi-valued ALC, if K is multi-valued consistent then there exists some finite multi-valued model of K.
- If K is multi-valued consistent then there exists some minimally multi-valued model of K. On the contrary, suppose that K has no minimally multi-valued models. Let $I_{1}$ be a multi-valued model of K since K has at least one multi-valued model. Then there would be an infinite sequence $\left\{I_{1},I_{2},\ldots \right\}$ of minimally multi-valued models for I such that $I{≻}_{\Sigma }I_{1}{≻}_{\Sigma }I_{2}{≻}_{\Sigma }\ldots$. We note that the number of concept names in Σ is finite since Σ is finite. By Definition 1 and Definition 2, there must exist some concept name *A* ∈ Σ such that ${\text{p}}^{+}\left(A^{I}\right)\cap {\text{p}}^{-}\left(A^{I}\right)$ is infinite. This is impossible as I is a finite model. In other words, every multi-valued model of K is infinite. Thus, we have arrived at a contradiction according to the first item.

*Proof of Proposition 14*. It directly follows Proposition 2 since we can assume that *φ* contains neither any concept name nor any role name occurring in K.

*Proof of Proposition 17*. If K is consistent then $Mod_{m_{c}}^{*}\left(K\right)=Mod_{m_{c}}\left(K\right)$. Because I is complete, ${\text{p}}^{+}\left(C^{I}\right)\cap {\text{p}}^{-}\left(C^{I}\right)=\Delta^{I}$ for any concept *C*. Assume that there exists some concept *C* and *a* ∈ Δ such that $a^{I}\in {\text{p}}^{+}\left(C^{I}\right)\cap {\text{p}}^{-}\left(C^{I}\right)$. Let $I^{\prime }$ be a complete base interpretation obtained as $C^{I^{\prime }}=\langle {\text{p}}^{+}\left(C^{I}\right)\setminus \left\{a^{I}\right\},{\text{p}}^{-}\left(C^{I}\right)\rangle$ (or $C^{I^{\prime }}=\langle {\text{p}}^{+}\left(C^{I}\right),{\text{p}}^{-}\left(C^{I}\right)\setminus \left\{a^{I}\right\}\rangle$). Because $I{⊨}_{m}C\left(a\right),I{⊨}_{m}\neg C\left(a\right)$ and K is consistent, we conclude that $I^{\prime }$ is also a complete multi-valued model of K. However, $I^{\prime }\;{≼}_{\Sigma }\;I$ but $I\neg \;{≼}_{\Sigma }\;I^{\prime }$ which contradicts I is minimally complete multi-valued model of K. Therefore, ${\text{p}}^{+}\left(C^{I}\right)\cap {\text{p}}^{-}\left(C^{I}\right)=\emptyset$.

*Proof of Proposition 18*. Let $K,K^{\prime }$ be two KBs.

1. We need to show that if $K{⊨}_{m}K^{\prime }$ then $K{⊨}_{m_{c}}K^{\prime }$. $K{⊨}_{m}K^{\prime }$ implies $Mod_{m}\left(K\right)\subseteq Mod_{m}\left(K^{\prime }\right)$ and $Mod_{m_{c}}\left(K\right)\subseteq Mod_{m}\left(K\right)$. For any $I\in Mod_{m_{c}}\left(K\right)$, $I\in Mod_{m}\left(K^{\prime }\right)$. Thus $I\in Mod_{m_{c}}\left(K^{\prime }\right)$ because I is complete. Then $Mod_{m_{c}}\left(K\right)\subseteq Mod_{m_{c}}\left(K^{\prime }\right)$. That is, $K{⊨}_{m_{c}}K^{\prime }$. However, let A={A(a),¬A⊔B(a)} be an ABox. Then, $A{⊨}_{m}^{min}B\left(a\right)$ while $A{⊭}_{m}B\left(a\right)$. Therefore, ${⊨}_{m}\;\subset \;{⊨}_{m}^{min}$.
2. We need to show that if $K{⊨}_{m}K^{\prime }$ then $K{⊨}_{m}^{min}K^{\prime }$. $K{⊨}_{m}K^{\prime }$ implies $Mod_{m}\left(K\right)\subseteq Mod_{m}\left(K^{\prime }\right)$ and $Mod_{m}^{min}\left(K\right)\subseteq Mod_{m}\left(K\right)$. Thus $Mod_{m}^{min}\left(K\right)\subseteq Mod_{m}\left(K^{\prime }\right)$. That is, $K{⊨}_{m}^{min}K^{\prime }$. However, let A={A(a),¬A⊔B(a)} be an ABox. Then $A{⊨}_{m}^{min}B\left(a\right)$ while $A{⊭}_{m}B\left(a\right)$. Therefore, ${⊨}_{m}\;\subset \;{⊨}_{m}^{min}$.
3. We need to show that if $K{⊨}_{m_{c}}K^{\prime }$ then $K{⊨}_{m_{c}}^{min}K^{\prime }$. $K{⊨}_{m_{c}}K^{\prime }$ implies $Mod_{m_{c}}\left(K\right)\subseteq Mod_{m_{c}}\left(K^{\prime }\right)$ and $Mod_{m_{c}}^{min}\left(K\right)\subseteq Mod_{m_{c}}\left(K\right)$. Thus $Mod_{m_{c}}^{min}\left(K\right)\subseteq Mod_{m_{c}}\left(K^{\prime }\right)$. That is, $K{⊨}_{m_{c}}^{min}K^{\prime }$. However, let A={A(a),¬A⊔B(a)} be an ABox. Then, $A{⊨}_{m_{c}}^{min}B\left(a\right)$ while $A{⊭}_{m_{c}}B\left(a\right)$. Therefore, ${⊨}_{m}\;\subset \;{⊨}_{m_{c}}^{min}$.

*Proof of Theorem 1*. We use Definition 9 and Proposition 4 to prove two directions of the theorem.

- (*soundness*) Assume that there exists an *m*_1_-open completion forest F∈forest(K). That is, for each node L(x)∈F, L(x) contains no **C2** clash in form of either $\left\{A,\bar{A}\right\}$ or $\left\{\neg A,\neg \bar{A}\right\}$.
  Next, we will show that an induced base interpretation I from F is a multi-valued model of K. That is, for any φ∈K, we have $I{⊨}_{m}\varphi$.
  1. *φ* = *R*(*a*, *b*). For any R(x,y)∈A, R∈L(〈x,y〉) in F. Thus if $a^{I}=x$, $b^{I}=y$ and $\left(x,y\right)\in {\text{p}}^{+}\left(R^{I}\right)$. Thus $I{⊨}_{m}R\left(a,b\right)$.
  2. *φ* = *C*(*a*). We will prove that C∈L(x) in F and $a^{I}=x$, then $I{⊨}_{m}C\left(a\right)$ by induction of subconcepts occurring in *C*.
    – Basic step: *C* = *A*, if $a^{I}=x$ then A∈L(x) in F. Thus $a^{I}\in {\text{p}}^{+}\left(A^{I}\right)$, that is, $I{⊨}_{m}A\left(a\right)$.
    – Induction step: assume that if $C_{i}\in L\left(x\right)$ in F and $a^{I}=x$, then $I{⊨}_{m}C_{i}\left(a\right)$ (*i* = 1, 2).
      - If $\neg C_{1}\in L\left(x\right)$ in F then $a^{I}\in {\text{p}}^{-}\left(C_{1}^{I}\right)$. Thus $I{⊨}_{m}\neg C_{1}\left(a\right)$.
      - If $\bar{C_{1}}\in L\left(x\right)$ in F then $a^{I}\notin {\text{p}}^{+}\left(C_{1}^{I}\right)$. Thus $I{⊨}_{m}{\bar{C}}_{1}\left(a\right)$.
      - If $\left(C_{1}⊓C_{2}\right)\in L\left(x\right)$ in F then $C_{1},C_{2}\in L\left(x\right)$ by the ⊓-rule. By assumption, $a^{I}\in {\text{p}}^{+}(C_{1}^{I}$ and $a^{I}\in {\text{p}}^{+}\left(C_{2}^{I}\right)$. Thus $I{⊨}_{m}C_{1}⊓C_{2}\left(a\right)$.
      - If $\left(C_{1}⊔C_{2}\right)\in L\left(x\right)$ in F then $C_{1}\in L\left(x\right)$ or $C_{2}\in L\left(x\right)$ by the ⊔-rule. By assumption, $a^{I}\in {\text{p}}^{+}\left(C_{1}^{I}\right)$ or $a^{I}\in {\text{p}}^{+}\left(C_{2}^{I}\right)$. Thus $I{⊨}_{m}C_{1}⊔C_{2}\left(a\right)$.
      - If $\forall R.C_{1}\in L\left(x\right)$ in F and R∈L(〈x,y〉) then $C_{1}\in L\left(y\right)$ by the ∀-rule. Thus if $\left(a^{I},b^{I}\right)\in {\text{p}}^{+}\left(R^{I}\right)$ and $b^{I}=y$ then $b^{I}\in {\text{p}}^{+}\left(C_{1}^{I}\right)$. Thus $I{⊨}_{m}\forall R.C_{1}\left(a\right)$.
      - If $\exists R.C_{1}\in L\left(x\right)$ in F then there exists some *y* such that R∈L(〈x,y〉) and $C_{1}\in L\left(y\right)$ by the ∃-rule. If $b^{I}=y$ then $\left(a^{I},b^{I}\right)\in {\text{p}}^{+}\left(R^{I}\right)$ and $b^{I}\in {\text{p}}^{+}\left(C_{1}^{I}\right)$. Thus $I{⊨}_{m}\exists R.C_{1}\left(a\right)$.
  3. *φ* = *C* ↦ *D*. By the second item of Proposition 4, for any base interpretation $I^{\prime }$, $I^{\prime }{⊨}_{m}C\mapsto D$ if and only if $I^{\prime }{⊨}_{m}\neg C⊔D\left(a\right)$ for all *a*. By the ↦-rule, for all node *x* in F, $\dot{\neg }C⊔D\in L\left(x\right)$. Thus we can use the proof of Item 2 (above) to prove that $I{⊨}_{m}\neg C⊔D\left(a\right)$ for all *a*.
  4. *φ* = *C* ⊏ *D*. By the third item of Proposition 4, for any base interpretation $I^{\prime }$, $I^{\prime }{⊨}_{m}C\;⊏\;D$ if and only if $I^{\prime }{⊨}_{m}\bar{C}⊔D\left(a\right)$ for all *a*. By the ↦-rule, for all node *x* in F, ∼C⊔D∈L(x). Thus we can use the proof of Item 2 (above) to prove that $I{⊨}_{m}\bar{C}⊔D\left(a\right)$ for all *a*.
  5. *φ* = *C* → *D*. By the forth item of Proposition 4, for any base interpretation $I^{\prime }$, $I^{\prime }{⊨}_{m}C\to D$ if and only if both $I^{\prime }{⊨}_{m}\bar{C}⊔D\left(a\right)$ and $I^{\prime }{⊨}_{m}\neg C⊔\neg \bar{D}\left(a\right)$ for all *a*. By the →-rule, for all node *x* in F, $\{∼C⊔D,\dot{\neg }C⊔\dot{\neg }\bar{D}\in L\left(x\right)$. Thus we can use the proof of Item 2 (above) to prove that $I{⊨}_{m}C\to D$ if and only if both $I{⊨}_{m}\bar{C}⊔D\left(a\right)$ for all *a*.
- (*completeness*) We need to show that if every completion forest F∈forest(K) is *m*_1_-closed then K has no any multi-valued model. Assume that I is a multi-valued model of K.
  Next, we use I to trigger the application of the multi-valued expansion rules such that they yield an *m*_1_-open completion F. To this purpose, a function *π* is inductively defined to map each node of F to an element of $\Delta^{I}$ such that, for each x,y∈nodes(F),
  $$\begin{array}{c} \begin{array}{c} \;\text{(i)}\;C\in L\left(x\right)\Rightarrow \pi \left(x\right)\in {\text{p}}^{+}\left(C^{}\right);\\ \;\text{(ii)}\;\text{if}\;y\;\text{is}\;\text{an}\;R\text{-child}\;\text{of}\;x,\;\text{then}\;\left(\pi {\left(x\right)}^{I},\pi {\left(y\right)}^{I}\right)\in {\text{p}}^{+}\left(R^{I}\right). \end{array} \end{array}$$ (4)
  It can be shown that the following claim holds:
  Let F be a completion forest and *π* be a function that satisfies Eq (4). If a multi-valued expansion rule is applicable to F, then this rule can be applied such that it yields a completion forest $F^{\prime }$ and a (possibly extended) *π* that satisfy Eq (4).
  We consider the various multi-valued expansion rules as follows:
  1. ⊓-rule: If $C_{1}⊓C_{2}\in L\left(x\right)$, then $\pi \left(x\right)\in {\text{p}}^{+}\left({\left(C_{1}⊓C_{2}\right)}^{I}\right)$. This implies $\pi \left(x\right)\in {\text{p}}^{+}\left(C_{1}^{I}\right)$ and $\pi \left(x\right)\in {\text{p}}^{+}\left(C_{2}^{I}\right)$, and hence the rule can be applied without violating Eq (4).
  2. ⊔-rule: If $C_{1}⊔C_{2}\in L\left(x\right)$, then $\pi \left(x\right)\in {\text{p}}^{+}\left({\left(C_{1}⊔C_{2}\right)}^{I}\right)$. This implies $\pi \left(x\right)\in {\text{p}}^{+}\left(C_{1}^{I}\right)$ or $\pi \left(x\right)\in {\text{p}}^{+}\left(C_{2}^{I}\right)$. Hence the rule can add a concept *C* ∈ {*C*_1_, *C*_2_} to L(x) such that $C\in L\left(x\right)\Rightarrow \pi \left(x\right)\in {\text{p}}^{+}\left(C^{I}\right)$ holds.
  3. ∃-rule: If ∃R.C∈L(x), then $\pi \left(x\right)\in {\text{p}}^{+}\left({\left(\exists R.C\right)}^{I}\right)$ and, there exists an element $t\in \Delta^{I}$ such that $\left(\pi \left(x\right),t\right)\in {\text{p}}^{+}\left(R^{I}\right)$ and $t\in {\text{p}}^{+}\left(C^{I}\right)$ since I is a multi-valued model of K. The application of ∃-rule generates a new variable *y* with L(〈x,y〉)={R} and L(y)={C}. Hence we set *π*: = *π*[*t* ← *y*] (i.e., *t* is replaced by *y*) which yields a function that satisfies Eq (4) for the modified forest.
  4. ∀-rule: If ∀R.C∈L(x), then $\pi \left(x\right)\in {\text{p}}^{+}\left({\left(\forall R.C\right)}^{I}\right)$ and, if *y* is an *R*-successor of *x*, then also $\left(\pi \left(x\right),\pi \left(y\right)\right)\in {\text{p}}^{+}\left(R^{I}\right)$ due to Eq (4). Because I is a multi-valued model of K, $\pi \left(y\right)\in {\text{p}}^{+}\left(C^{I}\right)$. Hence ∀-rule can be applied without Eq (4).
  5. ↦-rule: If C↦D∈T then for all x∈nodes(F), $\dot{\neg }\bar{C}⊔D\in L\left(x\right)$. Thus $\pi \left(x\right)\in {\text{p}}^{+}\left({\left(\dot{\neg }\bar{C}⊔D\right)}^{I}\right)$. Hence ↦-rule can be applied without Eq (4).
  6. ⊏-rule: If C ⊏ D∈T then for all x∈nodes(F), $\bar{C}⊔D\in L\left(x\right)$. Thus $\pi \left(x\right)\in {\text{p}}^{+}\left({\left(\bar{C}⊔D\right)}^{I}\right)$. Hence ⊏-rule can be applied without Eq (4).
  7. →-rule: If C→D∈T then for all x∈nodes(F), $\left\{\bar{C}⊔D,\dot{\neg }C⊔\dot{\neg }\bar{D}\right\}\subseteq L\left(x\right)$. Thus $\pi \left(x\right)\in {\text{p}}^{+}\left({\left(\bar{C}⊔D\right)}^{I}\right)$ and $\pi \left(x\right)\in {\text{p}}^{+}(\left({\left\{\dot{\neg }C⊔\dot{\neg }\bar{D}\right)}^{I}\right)$. Hence →-rule can be applied without Eq (4).
  For the initial completion forest consisting of a finite set of nodes *x*_*a*_ whose $L\left(x_{a}\right)=\left\{C\in clos\left(A\right)|C\left(a\right)\in A\right\}$ and $a^{I}=x_{a}$. We can give a function *π* satisfies Eq (4) by setting *π*(*x*_*a*_): = *s*_*a*_ for some $s_{a}\in \Delta^{I}$ with $s_{a}\in {\text{p}}^{+}\left(C^{I}\right)$ since I is a multi-valued model of K. Whenever a rule is applicable to $F_{K}$, it can be applied in a way that maintains Eq (4), and, must terminate by the analogous proof of [1]. Eq (4) implies that any completion forest F generated by these rule-applications must be open as there is only possibility for a **C2** clash in the following case, and it is easy to see that it can not hold in F: F can not contain a node *x* such that either $\left\{A,\bar{A}\right\}\in L\left(x\right)$ or $\left\{\neg A,\neg \bar{A}\right\}\in L\left(x\right)$ since for any A∈L(x), $\pi \left(x\right)\in {\text{p}}^{+}\left(A^{I}\right)$ and I is a multi-valued model of K.

2. To prove the second item of Theorem 1, we introduce induced complete base interpretation $I_{F}$ from F where for any concept *A*, ${\text{p}}^{+}\left(A^{I}\right)\cup {\text{p}}^{+}\left(A^{I}\right)=\Delta^{I}$.

We can use the analogous technique of the first item to prove the second item. For simplification, we show those differences here since complete multi-valued models are multi-valued models.

- (*soundness*) Assume that there exists an *m*_2_-open completion forest F∈forest(K). That is, for each node L(x)∈F, L(x) contains neither **C2** clash in form of either $\left\{A,\bar{A}\right\}$ or $\left\{\neg A,\neg \bar{A}\right\}$ nor **C3** clash in form of $\left\{\bar{A},\neg \bar{A}\right\}$.
  Next, we will show that an induced complete base interpretation I from F is a complete multi-valued model of K. That is, for any φ∈K, we have $I{⊨}_{m_{c}}\varphi$. We only need to show that $I_{F}$ is complete in the following cases: assume that ${\text{p}}^{+}\left(C_{i}^{I}\right)\cup {\text{p}}^{+}\left(C_{i}^{I}\right)=\Delta^{I}$ (*i* = 1, 2).
  1. *C* = ¬*C*_1_. Because ${\text{p}}^{+}\left(\neg C_{1}^{I}\right)={\text{p}}^{-}\left(C^{I}\right)$ and ${\text{p}}^{-}\left(\neg C_{1}^{I}\right)={\text{p}}^{+}\left(C^{I}\right)$, ${\text{p}}^{+}\left(\neg C_{1}^{I}\right)\cup {\text{p}}^{-}\left(C^{I}\right)=\Delta^{I}$.
  2. $C=\bar{C_{1}}$. ${\text{p}}^{+}\left({\bar{C_{1}}}^{I}\right)\cup {\text{p}}^{-}\left({\bar{C_{1}}}^{I}\right)=(\Delta^{I}\setminus {\text{p}}^{+}\left({\bar{C_{1}}}^{I}\right))\cup \Delta^{I}\setminus {\text{p}}^{-}\left({\bar{C_{1}}}^{I}\right))=\Delta^{I}\setminus \left({\text{p}}^{+}\left({\bar{C_{1}}}^{I}\right)\cap {\text{p}}^{-}\left({\bar{C_{1}}}^{I}\right)\right)=\Delta^{I}$ since ${\text{p}}^{+}\left({\bar{C_{1}}}^{I}\right)\cap {\text{p}}^{-}\left({\bar{C_{1}}}^{I}\right)=\emptyset$. Otherwise, assume that $a^{I}\in {\text{p}}^{+}\left({\bar{C_{1}}}^{I}\right)\cap {\text{p}}^{-}\left({\bar{C_{1}}}^{I}\right)$. Thus $\left\{\bar{C_{1}},\dot{\neg }\bar{C_{1}}\right\}\subseteq L\left(a^{I}\right)$. Then there exists some **C3** clash $\left\{\bar{A},\neg \bar{A}\right\}$ in some node of F which contradicts F is *m*_2_-open.
  3. *C* = *C*_1_ ⊓ *C*_2_. ${\text{p}}^{+}\left({\left(C_{1}⊓C_{2}\right)}^{I}\right)\cup {\text{p}}^{-}\left({\left(C_{1}⊓C_{2}\right)}^{I}\right)=\left({\text{p}}^{+}\left(C_{1}^{I}\right)\cap {\text{p}}^{+}\left(C_{2}^{I}\right)\right)\cup \left({\text{p}}^{-}\left(C_{1}^{I}\right)\cup {\text{p}}^{-}\left(C_{2}^{I}\right)\right)=\Delta^{I}\cap \Delta^{I}=\Delta^{I}$.
  4. *C* = *C*_1_ ⊔ *C*_2_. ${\text{p}}^{+}\left({\left(C_{1}⊔C_{2}\right)}^{I}\right)\cup {\text{p}}^{-}\left({\left(C_{1}⊔C_{2}\right)}^{I}\right)=\left({\text{p}}^{+}\left(C_{1}^{I}\right)\cup {\text{p}}^{+}\left(C_{2}^{I}\right)\right)\cup \left({\text{p}}^{-}\left(C_{1}^{I}\right)\cap {\text{p}}^{-}\left(C_{2}^{I}\right)\right)=\Delta^{I}\cap \Delta^{I}=\Delta^{I}$.
  5. *C* = ∃*R*.*C*_1_ and *φ* = ∀*R*.*C*_1_. We can conclude that ${\text{p}}^{+}\left({\left(\exists R.C_{1}\right)}^{I}\right)\cup {\text{p}}^{-}\left({\left(\exists R.C_{1}\right)}^{I}\right))=\Delta^{I}$ and ${\text{p}}^{+}\left({\left(\forall R.C_{1}\right)}^{I}\right)\cup {\text{p}}^{-}\left({\left(\forall R.C_{1}\right)}^{I}\right))=\Delta^{I}$.
  Therefore, for any *m*_2_ completion forest F, its induced base interpretation I from F is a multi-valued model of K by the proof of the first item of the theorem and we can show that I is complete. Thus K is complete multi-valued consistent.
- (*completeness*) Next, we need to prove that if every completion forest F in forest(K) is *m*_2_-closed then K has no any complete multi-valued model, i.e., multi-valued model. Assume that I is a complete multi-valued model of K. Analogously, for the initial completion forest consisting of a finite set of nodes *x*_*a*_ whose $L\left(x_{a}\right)=\left\{C\in clos\left(A\right)|C\left(a\right)\in A\right\}$ and $a^{I}=x_{a}$. We can give a function *π* satisfies Eq (4) by setting *π*(*x*_*a*_): = *s*_*a*_ for some $s_{a}\in \Delta^{I}$ with $s_{a}\in {\text{p}}^{+}\left(C^{I}\right)$ since I is a complete multi-valued model of K. Whenever a rule is applicable to $F_{K}$, it can be applied in a way that maintains Eq (4), and, must terminate by the analogous proof of [1].
  Eq (4) implies that any completion forest F generated by these rule-applications must be open as there are only two possibilities for clashes in the following cases, and it can not hold in F:
  1. (**C2** clash) F can not contain a node *x* such that either $\left\{A,\bar{A}\right\}\in L\left(x\right)$ or $\left\{\neg A,\neg \bar{A}\right\}\in L\left(x\right)$ since for any A∈L(x), $\pi \left(x\right)\in {\text{p}}^{+}\left(A^{I}\right)$ and I is a multi-valued model of K.
  2. (**C3** clash) F can not contain a node *x* such that $\left\{\bar{A},\neg \bar{A}\right\}\in L\left(x\right)$ since for any A∈L(x), $\pi \left(x\right)\in {\text{p}}^{+}\left(A^{I}\right)$ and I is a complete multi-valued model of K.

*Proof of Proposition 19*. By Definition 3, $\Delta^{I_{1}}=\Delta^{I_{2}}$ if $I_{1}\;{≼}_{\Sigma }\;I_{2}$, by Definition 7, $nodes\left(F_{1}\right)=nodes\left(F_{2}\right)$ if $F_{1}\;{⊴}_{\Sigma }\;F_{2}$ and Definition 9, $\Delta^{I_{1}}=nodes\left(F_{1}\right)$ and $\Delta^{I_{2}}=nodes\left(F_{2}\right)$.

On the one hand, if $F_{1}\;{⊴}_{\Sigma }\;F_{2}$, then for any node *x*, {A,¬A}∈L(x) in $F_{1}$ implies {A,¬A}∈L(x) in $F_{2}$ for any *A* ∈ *N*_*A*_ by Definition 7. Because $I_{1}⊨F_{1}$, by the definition, $x\in {\text{p}}^{+}\left(A^{I_{1}}\right)$ and $x\in {\text{p}}^{+}\left({\left(\neg A\right)}^{I_{1}}\right)$ implies $x\in {\text{p}}^{+}\left(A^{I_{1}}\right)\cap {\text{p}}^{-}\left(A^{I_{1}}\right)$. Because $I_{2}⊨F_{2}$, we can analogously conclude that $x\in {\text{p}}^{+}\left(A^{I_{2}}\right)$ and $x\in {\text{p}}^{+}\left({\left(\neg A\right)}^{I_{2}}\right)$ implies $x\in {\text{p}}^{+}\left(A^{I_{2}}\right)\cap {\text{p}}^{-}\left(A^{I_{2}}\right)$. Thus for any node *x*, $x\in {\text{p}}^{+}\left(A^{I_{1}}\right)\cap {\text{p}}^{-}\left(A^{I_{1}}\right)$ implies $x\in {\text{p}}^{+}\left(A^{I_{2}}\right)\cap {\text{p}}^{-}\left(A^{I_{2}}\right)$. Then, ${\text{p}}^{+}\left(A^{I_{1}}\right)\cap {\text{p}}^{-}\left(A^{I_{1}}\right)\subseteq {\text{p}}^{+}\left(A^{I_{2}}\right)\cap {\text{p}}^{-}\left(A^{I_{2}}\right)$. Moreover, there exists some node *x* such that {A,¬A}⊆L(x) in $F_{2}$ but {A,¬A}⊈L(x) in $F_{1}$ for some concept *A* ∈ *N*_*C*_. That is, ${\text{p}}^{+}\left(A^{I_{1}}\right)\cap {\text{p}}^{-}\left(A^{I_{1}}\right)\subset {\text{p}}^{+}\left(A^{I_{2}}\right)\cap {\text{p}}^{-}\left(A^{I_{2}}\right)$. Therefore, $I_{1}\;{≼}_{\Sigma }\;I_{2}$ by Definition 3.

On the other hand, if $I_{1}\;{≼}_{\Sigma }\;I_{2}$, for all concept name *A*, ${\text{p}}^{+}\left(A^{I_{1}}\right)\cap {\text{p}}^{-}\left(A^{I_{1}}\right)\subseteq {\text{p}}^{+}\left(A^{I_{2}}\right)\cap {\text{p}}^{-}\left(A^{I_{2}}\right)$. Because $I_{i}⊨F_{i}$ (*i* = 1, 2), for all *x*, by the definition, if $x\in nodes(F_{1})$ and $x^{I_{1}}\in {\text{p}}^{+}\left(A^{I_{1}}\right)\cap {\text{p}}^{-}\left(A^{I_{1}}\right)$ then $x^{I_{2}}\in {\text{p}}^{+}\left(A^{I_{2}}\right)\cap {\text{p}}^{-}\left(A^{I_{2}}\right)$ since $nodes\left(F_{1}\right)=nodes\left(F_{2}\right)$ and by the definition, all nodes in $F_{i}$ are taken as different individuals. Thus {A,¬A}⊆L(x) in $F_{1}$ implies {A,¬A}⊆L(x) in $F_{2}$. Therefore, $F_{1}\;{⊴}_{\Sigma }\;F_{2}$.

*Proof of Theorem 2*. We can use Proposition 4 and the first item of Theorem 1 and its results to prove this theorem. For simplification, we delete all details of the proof and instead, present a brief proof.

By Proposition 4 and the first item of Theorem 1, $K{⊨}_{m}C\left(a\right)$ if and only if $\text{forest}(K\cup \left\{\bar{C}\left(a\right)\right\}$ contains no *m*_1_-open completion forest.

- (soundness) If ${\text{forest}}^{min}\left(K\cup \left\{\bar{C}\left(a\right)\right\}\right)$ contains some *m*_1_-open completion forest F then there exists some multi-valued model I of $K\cup \{\bar{C}\left(a\right)\}$ by the proof of the first item of Theorem 1. By Proposition 19, $I\in Mod_{m}^{min}\left(K\right)$ and $I{⊨}_{m}\bar{C}\left(a\right)$ which contradicts $K{⊨}_{m}C\left(a\right)$.
- (completeness) assume that I is a minimally multi-valued model of K and a multi-valued model of $\bar{C}\left(a\right)$. Next, we use I to trigger the application of the multi-valued expansion rules such that they yield an *m*_1_-open minimally conflicting completion F. The process is the same as the proof of the first item of Theorem 1. Because all minimally multi-valued model of K are multi-valued models of K, thus I is a multi-valued model of $K\cup \{\bar{C}\left(a\right)\}$. By the previous proof, we can an *m*_1_-open forest F with I⊨F. By Proposition 19, $F\in {\text{forest}}^{min}\left(K\right)$ which contradicts the pre-condition.

*Proof of Theorem 3*. It analogously follows the second item of Theorem 1 and Proposition 19.

*Proof of Theorem 4*. Let $A^{*}=A\cup \left\{\bar{C}\left(a\right)\right\}$.

1. Firstly, we need to show that the problem of deciding whether $A{⊨}^{min}C\left(a\right)$ is PSPACE by employing the so-called *trace technique* in [1].
  - We denote $|A^{*}|$ as the size of $A^{*}$. Intuitively $|A^{*}|$ is the length required to write $A^{*}$ down, where we assume that the length required to write atomic concept, the negation of concept name and atomic role is “1”. Formally, we define the size of extended ABoxes as follows:
    $$\begin{array}{c} \begin{array}{ccc} |A^{*}| & = & \sum_{C\left(a\right)\in A^{*}}\left(|C|+1\right)+\sum_{R\left(a,b\right)\in A^{*}}3;\\ |A| & = & 1\;\text{for}\;\text{a}\;\text{concept}\;\text{name}\;A(\text{incl.}\;⊤,⊥);\\ |\neg D|=|\bar{D}| & = & |D|+1;\\ |D_{1}⊓D_{2}|=|D_{1}⊔D_{2}| & = & |D_{1}|+|D_{2}|+1;\\ |\exists R.D|=|\forall R.D| & = & |D|+2. \end{array} \end{array}$$
  - The multi-valued tableau algorithm generates a completion forest in a monotonic way. In a completion forest, for each individual name in $A^{*}$, the forest contains a root node, which we will call an *old node*. The edges between *old nodes* all stem from role assertions in $A^{*}$, and thus may occur without restrictions. Other nodes, called *new nodes*, are generated by the ∃-rule; we call the other rules *augmenting rules*, because they only augment the labels of existing nodes. In contrast to edges between old nodes, edges between new nodes are of a particular shape: each new node is found in a completion forest with an old node at its root.
  - Initially, for an old node *x*_*a*_, $L(x_{a})$ contains the concepts *D* from the assertions $a:D\in A^{*}$. Other concepts are added by the signed expansion rules, and we observe that these expansion rules only add subconcepts of the concepts occurring in $A^{*}$. Since there are at most $|A^{*}|$ such subconcepts, each node label can be stored in space polynomial in |*A*|. Moreover, for each concept $A^{*}$ in the label of a new node *x*, the (unique) predecessor of *x* contains a larger concept. Hence the maximum size of concepts in node labels strictly decreases along a path of new nodes, and thus the depth of each completion forest in our completion graph is bounded by $\max\{|D||a:D\in A^{*}\}$.
  - Finally, we note that the multi-valued expansion rules can be applied in an arbitrary order: the correctness proof for the algorithm does not rely on a specific application order. Hence we can use the following order: first, all augmenting rules are exhaustively applied to old nodes. Next, we treat each old node in turn, and build the tree rooted at it in a depth first manner. That is, for an old node *x*_*a*_, we deal in turn with each existential restriction $\exists R.D\in L(x_{a})$: we apply the ∃-rule in order to generate an *R*-successor *x*_0_ with $L\left(x_{0}\right)=\left\{D\right\}$, apply the ∀-rule exhaustively to this *R*-successor of *x*_*a*_ (which may add further signed concepts to $L(x_{0})$), and recursively apply the same procedure to *x*_0_, i.e., exhaustively apply the augmenting rules, and then deal with the existential restrictions one at a time.
  - On the other hand, a new completion forest will be added by applying the ⊔-rule one time. The $\left|forests(F_{A^{*}})\right|$ is in size linear in $|A^{*}|$ and the new nodes is in size linear in $|A^{*}|$. It is not hard to find that given two forests $F_{1},F_{2}$, checking if $F_{1}\;{⊴}_{\Sigma }\;F_{2}$ requires space polynomial in $|A^{*}|$. The problem of computing all minimally conflicting completion forests $forests_{min}\left(F_{A^{*}}\right)$ requires at most space polynomial in $|forests_{min}\left(F_{A^{*}}\right)|$ ≤ $\left|forests(F_{A^{*}})\right|$ and $|A^{*}|$. For each forest $F\in forests_{min}\left(F_{A^{*}}\right)$, if some node of F contains a **C2**-clash or **C3**-clash then we turn to check other forest. Thus we can investigate the *m*_1_-closed or *m*_2_-closed condition keeping a single branch in memory at any time. This branch is of length linear in $|A^{*}|$, and can thus be stored with all its labels in size polynomial in $|A^{*}|$. Therefore, continuing the investigation of all forests in the same manner, our algorithm only requires space polynomial in $|A^{*}|$.
2. Secondly, we can conclude that the problem deciding whether $A{⊨}^{min}C\left(a\right)$ is PSPACE-hard.
  The problem of deciding whether $A{⊨}_{m_{c}}C\left(a\right)$ is harder than that of problem of deciding whether $A{⊨}_{m}C\left(a\right)$, the problem of deciding whether $A{⊨}_{m}^{min}C\left(a\right)$ is harder than that of problem of deciding whether $A{⊨}_{m}C\left(a\right)$ and the problem of deciding whether $A{⊨}_{m_{c}}^{min}C\left(a\right)$ is harder than that of problem of deciding whether $A{⊨}_{m_{c}}C\left(a\right)$. Moreover, the problem of deciding whether $A{⊨}_{m}C\left(a\right)$ is equivalent to the problem of deciding whether $A{⊨}_{4}C\left(a\right)$ for any ABox A and any assertion *C*(*a*) in ALC which is PSPACE-hard stated in [21]. Thus the problem of deciding whether $A{⊨}_{p}C\left(a\right)$, where ⊨_*p*_ ∈ {⊨_*m*_, ⊨_*m*_*c*__, ⊨^*min*^} is PSPACE-hard.

Therefore, the problem of deciding whether $A{⊨}_{p}C\left(a\right)$, where ⊨_*p*_ ∈ {⊨_*m*_, ⊨_*m*_*c*__, ⊨^*min*^} is PSPACE-complete.

*Proof of Theorem 5*. Because the problem of deciding whether $\left(A,T\right){⊨}_{4}\varphi$ is ExpTime-complete [21], we only need to show that their problems are in ExpTime since their problems are hard than the four-valued entailment. We can employ the transformation algorithm, stated in [21] to reduce the four-valued entailment problem into the classical entailment by additionally transforming $\bar{A}$ into ¬*A* and then the multi-valued entailment problem can be reduced to the classical entailment. The complexity of the classical entailment in general ALC KBs is in ExpTime-complete [1]. The multi-valued entailment problem is ExpTime. Because given a set of completion forests, we can compute all conflicting completion forests in a polynomial time. Thus the minimally inconsistent entailment problem is also ExpTime.
